# Quantifying Efficiency Roll‐Off Factors in Quantum‐Dot Light‐Emitting Diodes

**DOI:** 10.1002/advs.202410041

**Published:** 2024-10-23

**Authors:** Xianchang Yan, Xitong Zhu, Boning Wu, Yizheng Jin, Wenming Tian, Shengye Jin

**Affiliations:** ^1^ State Key Laboratory of Molecular Reaction Dynamics Dalian Institute of Chemical Physics Chinese Academy of Sciences Dalian 116023 China; ^2^ University of Chinese Academy of Sciences Beijing 100049 China; ^3^ State Key Laboratory of Silicon and Advanced Semiconductor Materials Key Laboratory of excited‐ State Materials of Zhejiang Province, Department of Chemistry Zhejiang University Hangzhou 310027 China

**Keywords:** efficiency roll‐off, electrically pumped transient absorption spectroscopy, electron leakage, quantum dot light‐emitting diodes

## Abstract

The application of quantum‐dot light‐emitting diodes (QLEDs) is hindered by efficiency roll‐off at high current densities. Factors contributing to this roll‐off include Auger recombination, electric field‐induced quenching, Joule heating, and electron leakage into the hole transport layer. However, a method to quantitatively attribute the contribution of each factor to roll‐off has not yet been available, leaving the primary cause of roll‐off unidentified. This work addresses this gap using electrically pumped transient absorption spectroscopy, which measures the accumulated electrons and electric field in quantum dots (QDs). This study also introduces a method to quantify electron leakage in QLEDs using this spectroscopic technique. Based on the spectroscopic experimental results, the contribution of each factor to roll‐off is quantified. A green QLED with a peak external quantum efficiency (EQE) of 26.8% is studied as an example. The EQE declines to 20.5% at a current density of 354 mA cm^−2^, where field‐induced quenching accounts for 5% of the efficiency roll‐off, and electron leakage contributes 95%. Contributions from Auger recombination and heat‐induced quenching are negligible. This work demonstrates strong correlations between roll‐off and electron leakage amplitude using statistical data obtained in multiple QLEDs, confirming that electron leakage is the primary factor in EQE roll‐off.

## Graphical abstract

The contributions of Auger recombination, electric field‐induced quenching, and electron leakage to the efficiency roll‐off of QLEDs are quantified using electrically pumped transient absorption spectroscopy. The bleach signal, Stark effect signal, and leakage signal are used to calculate each of these factors, respectively. Electron leakage is proved to be the primary factor of EQE roll‐off.

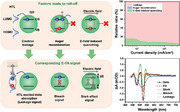

## 1 Introduction

Quantum‐dot light‐emitting diodes (QLEDs) have emerged as the next generation of electroluminescent devices, favored for their high color purity, low‐cost fabrication, and high efficiency.^[^ ^1^, ^2^, ^3^, ^4^, ^5^, ^6^, ^7^, ^8^, ^9^, ^10^, ^11^, ^12^, ^13^, ^14^, ^15^ ^]^ The latest bottom‐emitting QLEDs have achieved remarkable external quantum efficiencies (EQEs) of 35.6% for red, 28.7% for green, and 23% for blue devices, approaching their respective theoretical limits.^[^ ^16^, ^17^, ^18^ ^]^ If top‐emission designs or tandem structures are adopted, even higher EQEs could be achieved.^[^ ^19^, ^20^ ^]^ However, these impressive EQEs are typically achieved at low currents. With increased current flow leading to enhanced QLED luminescence, the EQE decreases, a phenomenon known as efficiency roll‐off or droop.^[^ ^21^, ^22^ ^]^ This effect poses a challenge for specific QLED applications, such as lighting and outdoor displays, which require both high brightness and superior efficiency. Pursuing QLEDs with high efficiency at high brightness has emerged as a key area of focus in recent research. Therefore, it is crucial to elucidate the factors driving the efficiency roll‐off observed in QLEDs.^[^ ^23^, ^24^ ^]^

**Figure 1:**
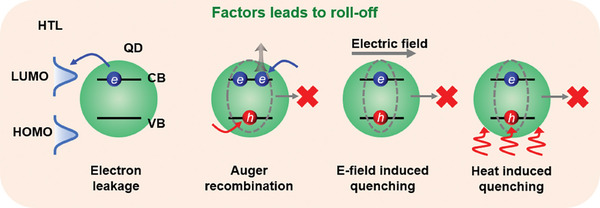
Factors leading to EQE roll‐off in QLEDs, including electron leakage from the QDs into the HTL, Auger recombination, electric field induced quenching, and Joule heat induced quenching.

Currently, the underlying mechanism of efficiency roll‐off in QLED remains under debate.^[^ ^22^, ^25^ ^]^ Significant factors identified as contributing to roll‐off include nonradiative Auger recombination,^[^ ^14^, ^26^, ^27^ ^]^ electron leakage,^[^ ^25^, ^28^ ^]^ exciton quenching induced by electric field (E‐field),^[^ ^29^ ^]^ and quenching by Joule heat.^[^ ^13^, ^30^ ^]^ These four factors are illustrated in **Figure** **1**. Auger recombination is a nonradiative process in which the energy released from the recombination of an electron‐hole pair is transferred to a third carrier instead of being emitted as a photon. In a QLED, the electron injection barrier is lower than that of holes, leading to faster electron injection and subsequent electron accumulation within the quantum dot (QD) layer. This accumulation facilitates the Auger recombination processes that two electrons recombine with one hole. In conventional bulk semiconductor light‐emitting diodes, the impact of Auger recombination on roll‐off can be analyzed using the well‐known ABC model.^[^ ^31^, ^32^, ^33^ ^]^ However, in QLEDs, where carriers are confined within QDs, quantum confinement effects and Coulomb charging energy between carriers must be taken into account. Therefore, the ABC model becomes unsuitable, requiring an alternative calculation method.^[^ ^34^ ^]^ Electron leakage is the tunneling of electrons in the conduction band (CB) of the QDs into the Lowest Unoccupied Molecular Orbital (LUMO) of the hole transport layer (HTL), also facilitated by the accumulation of electrons, which elevates the Fermi level of the QDs.^[^ ^17^ ^]^ Electric field‐induced quenching refer to the reduction of the photoluminescence quantum yield (PLQY) of the QDs in the QLED under electric field. Joule heating, primarily arising from nonradiative recombination in QDs or the HTL, can reduce the PLQY of QDs and potentially damage the HTL.

It remains unclear which of these factors predominantly causes EQE roll‐off, as there is no method to directly measure their individual contributions toward roll‐off. The goal of this work is to fill this gap. This is achieved through our recently developed electrically pumped transient absorption (E‐TA) technology.^[^ ^35^ ^]^ This technology can measure the number of accumulated electrons in the QDs and the magnitude of the electric field across the QD layer during operation, enabling us to quantify the contributions of Auger recombination and electric field‐induced quenching. Additionally, in this work, we discovered that the technology also allows us to quantify the level of electrons in the HTL that have leaked from the QDs, facilitating the study of electron leakage. Thus, all factors contributing to roll‐off can be quantified through E‐TA technology.

## 2 Results and Discussions

### 2.1 Introduction to E‐TA Spectroscopy and the Leakage Signal

**Figure 2:**
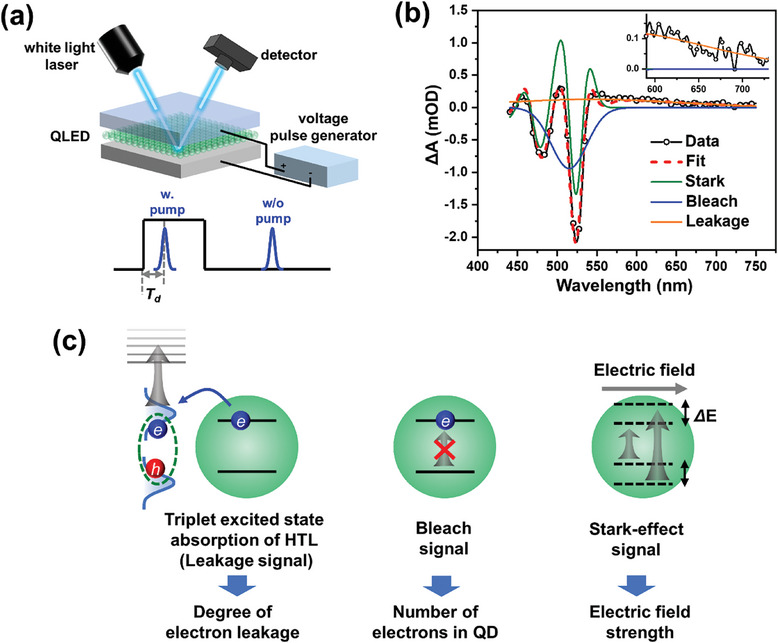
a) The structure of the E‐TA spectroscopy. b) E‐TA spectrum of a green QLED pumped at 4 V voltage (354 mA cm^−2^ of current density), decomposed into distinct signals: electron bleach, Stark effect, and a broad band attributed to electron leakage. The inset shows a zoomed‐in view of the graph to emphasize the leakage signal. c) Interpretation of the E‐TA leakage signal (excited state absorption of excitons in HTL), bleach signal (due to electron occupation of CB of QDs), and the Stark‐effect signal (caused by quantum confined Stark effect of the QDs), and the information that can be obtained from these signals.

The configuration of the E‐TA setup is shown in **Figure** **2**a. This method involves applying square voltage pulses to illuminate the QLEDs and using white light laser pulses to probe changes in absorbance (ΔA) of the QLED upon electric excitation. The time delay (T_d_) between the laser pulse and the rising edge of the voltage pulse is adjustable, enabling the system to track changes in the absorption spectra over time. Figure 2b showcases an example E‐TA spectrum of a green QLED at a pump voltage of 4 V with T_d_ = 9000 ns. The spectrum can be deconstructed into three components: a negative Gaussian‐shaped peak centered around the emission wavelength (bleach signal), a multi‐lobed peak (Stark effect signal), and a broad peak at the red edge (leakage signal). Such deconstruction is achieved using a global fit on the E‐TA spectra acquired at various T_d_ and voltages. Details of the fitting process are provided in Figures S8–S11, Supporting Information. The physical interpretation and insights obtainable from these three types of signals are elucidated in Figure 2c. The bleach signal arises from electrons occupying the CB of the QDs, providing a means to assess the average number of accumulated electrons in each QD (*N_e_*).^[^ ^35^ ^]^ The Stark effect signal corresponds to the quantum‐confined Stark effect of the QDs and serves as an indicator for evaluating the intensity of the electric field. More details about the Stark and bleach signals are available in our previous work.^[^ ^35^ ^]^ In this study, we discover the leakage signal and identify it as the triplet excited‐state absorption of the HTL, which results from the recombination of electrons that leaked into the HTL with pre‐existing holes. Therefore, the intensity of the leakage signal is proportional to the number of electrons that leak into the HTL from the QDs, serving as a measure of electron leakage. Comprehensive verification of the signal's nature is provided in the Supporting Information (Figures S2–S5, Supporting Information).^[^ ^36^, ^37^, ^38^ ^]^

### 2.2 Quantifying the Impact of Each Roll‐Off Factor Using E‐TA Spectroscopy

**Figure 3:**
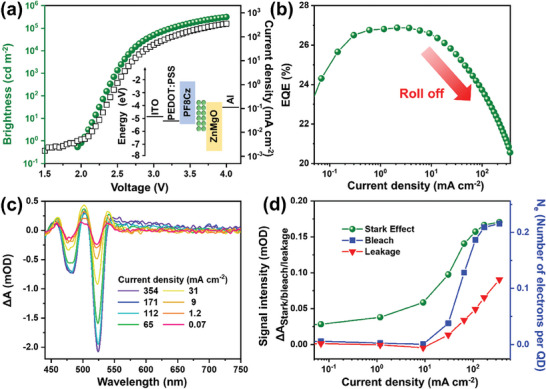
a) The brightness and current of a green QLED as a function of voltage, with the QLED structure inset. b) EQE of the QLED as a function of current density. c) Equilibrium E‐TA spectra at different corresponding current densities, taken where T_d_ > 8 µs. d) Amplitude of Stark, bleach, and leakage signals (ΔA_Stark/bleach/leakage_) as a function of current density, obtained after decomposing the spectra into three types of signals. The right axis illustrates the value of *N_e_* (number of electrons per QD) that is correlated with bleach signal by a linear relationship that *N_e_* = *α* × ΔA_bleach_. For this specific QLED, the constant *α* = 1280. The signal amplitude is the average of absolute value (|ΔA|) of the signal after extracted from the total spectrum (as shown in Figure 2b), within the range of 440 to 750 nm. Additional details on calculating ΔA_Stark/bleach/leakage_ from E‐TA spectra and obtaining the correlation between *N_e_* and ΔA_bleach_ are provided in Figures S8–S10 and S13, Supporting Information, respectively.

We evaluate the efficiency roll‐off factors in a green QLED as an example.^[^ ^17^ ^]^ **Figure** **3**a,b shows the device luminescence and current as functions of voltage, and its EQE as a function of current density. This QLED has a peak EQE of 26.8% at 2.1 mA cm^−2^. As the current density increases to 354 mA cm^−2^, the EQE declines to 20.5%. The relative efficiency, denoted as *η*_EQE_(ρ), as a function of current density, is calculated by dividing the EQE at a specific current density ρ by the peak EQE value. Assuming that no additional factors beyond the four presented here significantly influence efficiency roll‐off, we can express *η*_EQE_(ρ) as the product of the residual efficiencies after accounting for electric field‐induced quenching (*η*_E‐field_), Auger recombination (*η*_Auger_), electron leakage (*η*_leak_), and Joule heat‐induced quenching (*η*_heat_), respectively:

(1) $$\begin{array}{c} \eta_{EQE}\left(\rho \right)=\frac{EQE(\rho )}{EQE(\max)}=\eta_{E-field}\left(\rho \right)\times \eta_{Auger}\left(\rho \right)\times \eta_{leak}\left(\rho \right)\times \eta_{heat}\left(\rho \right) \end{array}$$

**Figure 4:**
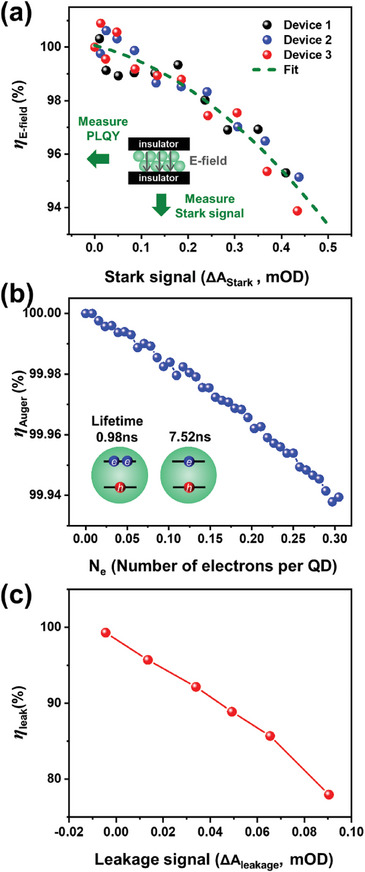
The correlation between the relative EQE and the intensity of Stark, leakage signals, and the number of electrons per QD. a) The residual efficiency after accounting for *η*_E‐field_ as a function of ΔA_Stark_. To obtain this curve, we measure Stark effect signal intensity and relative PLQY of the QDs at the same applied voltage, where Stark signal intensity goes to x‐axis and PLQY goes to y‐axis. Applying voltage to QD layer without carrier injection is achieved by fabricating specialized devices, where QDs are sandwiched between two insulator layers. Three distinct specialized devices are tested and represented with dots of different colors, and the fit is presented as a dashed curve. b) *η*_Auger_ as a function of *N_e_* (*N_e_* ∝ ΔA_bleach_), computed according to Equation (2). c) The correlation between *η*_leak_ and ΔA_leakage_.

The aim of this work is to precisely quantify each *η* component in Equation (1) in the operating QLED, thereby clarifying the key factors influencing efficiency roll‐off. The first step is to measure the intensity of the bleach signal (ΔA_bleach_(ρ)), Stark signal (ΔA_Stark_(ρ)), and leakage signal (ΔA_leakage_(ρ)) as functions of current density using E‐TA experiments. This is discussed in Section 2.2.1 and illustrated in Figure 3. The second step is to seek the precise correlations between ΔA_Stark_ and *η*_E‐field_, between ΔA_bleach_ and *η*_Auger_, as well as between ΔA_leakage_ and *η*_leak_, which is discussed in Section 2.2.2 and shown in **Figure** **4**. The correlation between ΔA_Stark_ and *η*_E‐field_, as well as between ΔA_bleach_ and *η*_Auger_, is determined through experimental methods or theoretical calculations. *η*_leak_ is derived from the residual values of the other *η* factors, expressed as *η*_leak_ = *η*_EQE_ / (*η*_E‐field_ • *η*_Auger_ • *η*_heat_). We then examine the correlation between ΔA_leakage_ and *η*_leak_ to assess the impact of current leakage on efficiency roll‐off. Finally, the signal intensities obtained in Seciton 2.2.1 are substituted into the correlations obtained in the Section 2.2.2 to calculate *η*(ρ) for each roll‐off factor as a function of current density.

We omit the influence of Joule‐heat induced quenching in this study, as previous research indicates its significance only at exceptionally high currents (> 2500 mA cm^−2^).^[^ ^13^, ^30^ ^]^ To confirm this, we measure the temperature of the QLED at different operating currents. At 400 mA cm^−2^, the temperature rise is less than 7 °C, a change at which QD efficiency reduction is negligible (see Figure S6, Supporting Information).^[^ ^13^, ^39^, ^40^ ^]^ Therefore, we can comfortably consider *η*_heat_ as 1, focusing solely on the influence of the other three factors.

#### 2.2.1 Measuring Bleach, Stark, and Leakage Signal Intensities in an Operating QLED

Figure 3c shows the equilibrium E‐TA spectra of the QLED under different current densities. The spectra are decomposed into the three signals, including bleach, Stark effect, and leakage signals, as illustrated in Figure 2b, by applying a global fit. More details are available in Figures S8–S11, Supporting Information.

Decomposition of E‐TA spectra at different T_d_ is detailed in Figure S8, Supporting Information, resulting in time‐dependent data for the three signal types (Figure S9, Supporting Information). For analyzing the roll‐off factors, we only use the equilibrium signal intensities at T_d_ between 8 and 10 µs. Figure S10, Supporting Information reveals the decomposition of E‐TA spectra at different voltages, and the time dependence of each signal type at different pump voltages is displayed in Figure S11, Supporting Information.

The amplitude of each signal (ΔA_Stark/bleach/leakage_) plotted against current is shown in Figure 3d. These intensities represent the average absolute value (|ΔA|) of the decomposed spectra for a specific signal across the 440 to 750 nm range. Among these, ΔA_bleach_ is proportional to *N_e_* (the average number of electrons per quantum dot) by *N_e_* = *α* × ΔA_bleach_. The constant *α* is equal to 1280 for the QLED presented. The right axis of Figure 3d shows the *N_e_* value for the correlated bleach signal. Method for converting the bleach signal intensity to *N_e_* value is available in Figure S13, Supporting Information.^[^ ^41^ ^]^

#### 2.2.2 Establishing the Correlation between EQE Roll‐Off and E‐TA Signal Intensities

**Figure** **4**a–c illustrates the correlations between *η*_E‐field_ and ΔA_Stark_, between η_Auger_ and *N_e_* (*N_e_* ∝ ΔA_bleach_), as well as between *η*_leak_ and ΔA_leakage_, respectively. Correlation between *η*_E‐field_ and ΔA_Stark_ is derived from measuring the relative PLQY of QDs under varying electric field intensities in customized devices. These devices contain a QD layer sandwiched between two insulator layers of polymethyl methacrylate (PMMA), which prevents carrier injection into the QDs. E‐TA spectra, containing Stark effect signal only, from these customized devices are measured at varying pump voltages and ΔA_Stark_ are obtained at each pump voltage. The PLQY of the insulative devices, under identical external voltages, is also measured using both steady‐state and time‐resolved fluorescence methods with laser excitation. The combination of these measurements allows for plotting PLQY against ΔA_Stark_ in Figure 4a. Consistent results are observed across three individual customized devices, each represented by a different color. Polynomial fitting yields a curve representing the relationship between *η*_E‐field_ and ΔA_Stark_, depicted by the dashed line. Details of this measurement, including the E‐TA spectra, PL spectra, and PL lifetime of the specialized devices, can be found in Figure S12, Supporting Information.

Figure 4b illustrates the relationship between ηAuger and Ne. When Auger recombination occurs, there are two potential scenarios. The first involves an electron entering an excited QD before its emission. However, our calculation reveals that at 354 mA cm^−2^ current density, an electron enters a QD every 520 ns on average, a time notably greater than the lifetime of the QD exciton. (A relationship between electron injection time interval and current density can be found in Figure S14a, Supporting Information.) Therefore, it is unlikely for an exciton to encounter another electron. The second scenario involves a hole entering a QD^2−^. In this case, *η*_Auger_ is calculated as:

(2) $$\begin{array}{ccc} \eta_{Auger} & = & \frac{P\left(1\right)\Phi_{X}+P\left(2\right)\Phi_{X^{-}}}{\Phi_{X}}\\ & = & P\left(1\right)+P\left(2\right)\Phi_{X^{-}}/\Phi_{X}=P\left(1\right)+P\left(2\right)\beta \tau_{X^{-}}/\tau_{X} \end{array}$$

In this equation, X and X^−^ denote a neutral exciton and a negative trion in the QD, respectively. P(1) and P(2) represent the proportion of QDs containing 1 or 2 electrons among all negatively charged QDs. Given the degeneracy of QD 1S orbital, we consider a QD can contain only 0,1 or 2 electrons. Φ and τ depict the PLQY and lifetimes of an excited species, respectively. β is the ratio between radiative decay rates of a negative trion and an exciton, which is equal to 1.5 for this type of QDs.^[^ ^42^, ^43^ ^]^The PL lifetime of X and X^−^ (τ_*X*_ and $\tau_{X^{-}}$) are 7.52 and 0.98 ns, respectively, as measured via time‐resolved fluorescence methods. Additional details can be found in Figure S14b, Supporting Information.^[^ ^42^, ^43^ ^]^

P(2) is dependent on *N_e_* and the electron charging energy (E_c_), where E_c_ represents the additional energy required for an electron to enter a QD already occupied by another electron. For the QDs we used in this study, E_c_ is estimated at 150 meV.^[^ ^44^, ^45^ ^]^ The value of P(2) is simulated using a Monte Carlo method. Starting with an electron‐free QD layer, electrons are incrementally added to random QDs up to a total occupancy of *N_e_*. At this time, P(2) represents the fraction of QDs containing 2 electrons within all occupied QDs. During the simulation, the probability of an electron entering an empty QD is greater than that entering a single‐electron‐occupied QD by a factor of 𝑍, where 𝑍 is the partition function given by Z = exp(E_c_​/*k_B​_T*). Here, 𝑘_𝐵_​ is the Boltzmann constant, and 𝑇 is the temperature. More details and results regarding the calculation of P(2) are provided in Figure S15, Supporting Information.^[^ ^46^, ^47^ ^]^

η_leak_, calculated as the residual of *η*_EQE_ after dividing by *η*_E‐field_ and *η*_Auger_, is examined with ΔA_leakage_, as depicted in Figure 4 (c). A strong relationship is observed, validating our method of η_leak_ calculation. We will further discuss the influence of electron leakage on efficiency roll‐off in Section 2.3.

#### 2.2.3 Assessing the Impact of Roll‐Off Factors in an Operating QLED

**Figure 5:**
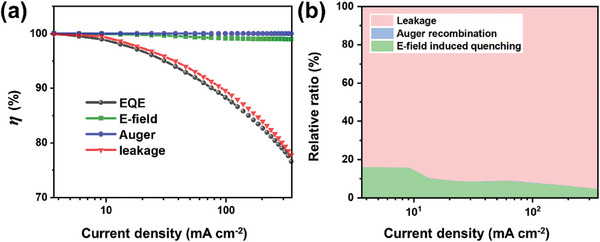
a) The residual efficiencies of the QLED, after accounting for *η*_E‐field_, *η*_Auger_, and *η*_leak_, respectively, plotted alongside *η*_EQE_. *η*_heat_ is estimated to be always equal to 1. b) A visualization of the percentage contribution of each of these three factors toward EQE roll‐off. The contribution of heat induced quenching is neglected.

The signal intensities obtained in Section 2.2.1 are substituted into the correlations obtained in Section 2.2.2 to calculate *η*(ρ) for each roll‐off factor as a function of current density. **Figure** **5**a shows our final results, where *η*_E‐field_, *η*_Auger_, and *η*_leak_ are plotted alongside *η*_EQE_, with *η*_heat_ neglected. Figure 5b provides a rough visualization of the contribution of each EQE roll‐off factor. For example, the fraction of electric field‐induced quenching is represented by (1 – *η*_E‐field_) / (1 – *η*_E‐field_ + 1 – *η*_Auger_ + 1 – *η*_leak_), with similar calculation for Auger recombination and electron leakage contributions. At a current density of 354 mA cm^−2^, the EQE decreases to 20.5% from its peak value of 26.8%. The Stark effect contributes roughly 5% to the roll‐off at this current, whereas electron leakage accounts for a significant 95%. The contribution from Auger recombination is negligible, due to the small population of QD^2−^ at the measured *N_e_* value. Given an E_c_ of 150 meV, it is unlikely for an electron to enter an already negatively charged QD. We reassess the contribution of Auger recombination assuming a considerably lower E_c_ = 30 meV in Figure S16, Supporting Information. Even in this scenario, the contribution of Auger recombination remains minimal.

### 2.3 Further Verification of the Correlation between Roll‐Off and Electron Leakage

**Figure 6:**
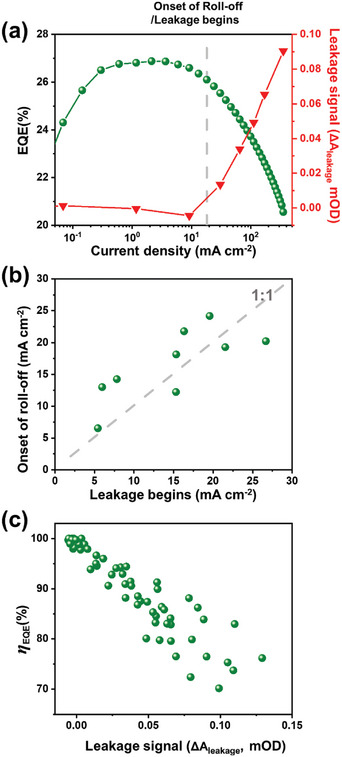
Additional evidences indicating that EQE roll‐off is dominated by electron leakage. a) The amplitude of leakage signal and EQE as a function of current density in a QLED. b) The correlation between the initiation current density of roll‐off and the current density at which the leakage signal emerges, analyzed across nine individual QLEDs, with each dot representing a device. The dashed grey line is a 1:1 reference line. c) The correlation between the amplitude of the leakage signal and *η*_EQE_, obtained from statistical analysis of 9 QLED devices. Each QLED contributes multiple points to this graph at different current densities.

According to Figure 5, electron leakage is the primary factor of QLED EQE roll‐off. To further substantiate this claim, we provide two additional pieces of evidence by investigating the correlation between the leakage signal and efficiency roll‐off in **Figure** **6**. First, in the same QLED, when the current density increases, the current density where leakage signal begins to be observed coincides with that roll‐off begins. In Figure 6a, both EQE and the leakage signal amplitude are plotted against current density. Up to 0.1 mA cm^−2^, the EQE increases with the current as trap states in the QD become filled.^[^ ^48^ ^]^ Once the peak is reached, the EQE plateaus briefly before starting to decrease with further increases in current density, coinciding with the onset of the leakage signal. This experiment, consistently repeated in nine QLEDs, shows a fitting trend along a 1:1 in Figure 6b when comparing the point where the leakage signal emerges (intercepted where ΔA_leakage_ > 0.002 mOD) against the onset of roll‐off (defined as EQE reduced to 98% of its peak value). Second, we observe a strong correlation between *η*_EQE_ and ΔA_leakage_. As demonstrated in Figure 6c, a robust correlation between ΔA_leakage_ and the degree of roll‐off is clearly observed across 9 QLEDs at various current densities. The comparison between EQE and ΔA_leakage_ in each QLED device are available in Figures S18 and S19, Supporting Information. Unlike the strong correlation between *η*_EQE_ and ΔA_leakage_, no significant correlation is found between *η*_EQE_ and ΔA_bleach_ or ΔA_Stark_, further confirming that leakage is the dominant factor in roll‐off, as illustrated in Figures S20 and S21, Supporting Information. We also examine the correlation between *η*_EQE_ and ΔA_leakage_ in blue QLEDs, where similar correlations are observed, as shown in Figure S22, Supporting Information. Therefore, we conclude that electron leakage is the primary factor influencing EQE roll‐off in QLEDs.

## 3 Conclusions

In this study, we successfully quantified the factors contributing to efficiency roll‐off in a QLED, including electric‐field induced quenching, Auger recombination, and electron leakage. That is achieved through the use of our newly developed E‐TA technology. This technology enables the quantification of the electric field across the QD layer via a Stark effect signal, the number of accumulated electrons in the QDs via a bleach signal, and the degree of electron leakage via the excited state absorption signal of the HTL (referred to as the leakage signal).

In a typical green QLED, we observed a peak EQE of 26.8% at a low current density of 2.1 mA cm^−2^, which declined to 20.5% at a current density of 354 mA cm^−2^. At this point, contribution to roll‐off was approximately at 5% from the electric field inducing quenching and 95% from the electron leakage, with negligible contributions from Auger recombination and heat induced quenching. To confirm electron leakage as the primary factor in EQE roll‐off, we investigated its relationship with the amplitude of the leakage signal. We observed that the initiation of the leakage signal coincided with the onset of EQE roll‐off and discovered a strong correlation between the amplitude of the leakage signal and EQE roll‐off.

Our findings indicate that reducing electron leakage is crucial for mitigating efficiency roll‐off in QLEDs. Strategies to achieve this objective may involve optimizing the QD structure, enhancing hole injection for improved charge balance, elevating the LUMO level of HTL material, and minimizing energy disorder in the HTL. It should be noted that this conclusion may vary with different types of QLEDs, especially if there are alterations to the QD structure or a failure in achieving proper charge balance. These changes could potentially amplify the impact of Auger recombination or electric field‐induced quenching.

## 4 Experimental Section

### Fabrication of QLEDs and Customized Devices

QLEDs of structure ITO/PEDOT: PSS/PF8Cz/QDs/Zn0.85Mg0.15O/Al:

ITO‐coated glass substrates (≈20 Ω sq^−1^) were cleaned using ultrasonic cleaning and oxygen plasma for 10 min. A substrate was spin‐coated with a PEDOT:PSS solution (Baytron PVP Al 4083) at 3000 rpm for 40 s and then baked at 150 °C for 30 min before being transferred into a nitrogen‐filled glovebox, where both oxygen and water levels were controlled below 1 ppm. The Hole Transport Layer (HTL) was formed by spin‐coating PF8Cz (8 mg mL^−1^ in chlorobenzene) at 2000 rpm for 40 s, followed by baking at 150 °C for 30 min. The green QDs that was used had a structure of CdSe/CdZnSe/ZnSe with a diameter of ≈10 nm, while the blue QDs have a structure of CdZnSe/ZnS with a diameter of ≈10.2 nm. Solutions of QD (≈15 mg mL^−1^ in octane) and Zn_0.85_Mg_0.15_O nanocrystals (≈23 mg mL^−1^ in ethanol) were then spin‐coated sequentially at 2000 rpm for 30 s and baked at 85 °C for 30 min. Finally, the devices were transferred into a high vacuum (≈10^−7^ torr), and Al electrodes, 100 nm thick, were deposited via thermal evaporation (Trovato 300 C). The device area was 4 mm^2^, defined by the overlapping of the ITO and metal electrodes. The devices were encapsulated with ultraviolet‐curable resins in a nitrogen‐filled glovebox.

Specialized devices with structures ITO/polymethyl methacrylate (PMMA)/QDs/PMMA/Al

The first layer of PMMA (between ITO and QDs) was prepared by spin‐coating a solution of PMMA (10 mg mL^−1^ in in ethyl acetate) onto ITO‐coated substrates at 2000 r.p.m. for 30 s and baked at 120 °C for 15 min. The second layer of PMMA (between QDs and Al) was spin‐coated onto the QD films using the same parameters, without baking. The QD layer and Al electrode were made using the same procedure as in QLEDs.

Specialized devices with structures ITO/PEDOT: PSS/PF8Cz/PMMA/Al, ITO/LiF/PF8Cz/Zn_0.85_Mg_0.15_O/Al, and ITO/LiF/PF8Cz/PMMA/Al

PMMA layers were made by spin‐coating PMMA solutions (10 mg mL^−1^ in ethyl acetate) at 2000 rpm for 30 s. LiF layers of 40 nm thick were deposited by thermal evaporation (Trovato 300 C) under high vacuum (≈10^−7^ torr). Other layers were made with the same procedure as in QLEDs.

### EQE Measurement

The voltage‐current‐luminescence curve was obtained through the following procedure: A digital source meter (Keithley 2400) applies DC voltage to the QLED. The electroluminescence emission was then collected by an integration sphere (3P‐GPS‐033, Labsphere). The emission was detected by a spectrometer (QEpro, Ocean Optics). EL and current are calibrated using a luminous flux calibration halogen light source (Labsphere).

### Quantum Yield Measurements

This study measured the PL spectra and lifetimes of the QDs to assess their relative quantum yield, either under the influence of electric fields or in the case of negatively charged. A 450 nm excitation pulse was generated using a supercontinuum white light fiber laser (SuperK FIANIUM, NKT Photonics), with the wavelength selected via an Acousto‐Optical Tunable Filter system (Fianium). PL spectra were acquired using a high‐performance spectrometer (HRS‐300‐S, Teledyne Princeton Instruments), while time‐resolved PL was measured using a time‐correlated single photon counting (TCSPC) module (SPC‐150, Becker & Hickl GmbH).

### Electric‐Pumped Transient Absorption

In summary, the E‐TA equipment delivered square voltage pulses to illuminate the QLEDs and used white light laser pulses to probe their absorbance. The white light probe laser penetrated the ITO surface, the QDs, and carrier transport layers, then reflected off the metal electrode, thus probing the absorption spectra of the QLED. The time delay (T_d_) between the laser pulse and the rising edge of the voltage pulse was adjusted to measure the absorption spectra as a function of time. During each cycle of the voltage pump pulse, two probe light pulses were sent to measure the absorbance both with and without the pump voltage. The change in absorbance (ΔA) was determined by subtracting the two measurements. To eliminate the contribution of electroluminescence (EL) to the spectra, the ΔA spectrum where T_d_ < 0 is subtracted as a background, since the detector integrated the collected light over all time, ensuring the entire EL emission was collected for each T_d_ before subtracted.

The power source was an arbitrary waveform generator (Tektronix AFG31002), which generated voltage pulses with a frequency of 1 kHz and rising/falling edges of less than 4 ns. Along with the voltage pulse, it generated an electronic trigger signal to trigger a supercontinuum laser (LEUKOS DISCO‐2, 2 kHz) that produced white probe laser light with a frequency of 2 kHz and 50 nJ per pulse. The time delay T_d_ between the probe laser and voltage pulse was added to the electric trigger signal by a time delay generator (PCIe‐6612, National Instruments). To remove timing jitter in the system, the actual value of T_d_ was recalculated by comparing the arrival times of the voltage pulse and white laser using a Pendulum CNT‐91 frequency analyzer. Therefore, the resolution of the technology was reduced to ≈1 ns. The T_d_ = 0 point was determined by replacing the QLED with a photodiode (DET10A2, Thorlabs) to measure the white light arrival time.

To avoid laser damage, the probe beam was not focused. To improve the signal‐to‐noise ratio, a reference detector calibrated the intensity fluctuations of the probe laser beam. The reference beam was split from the probe light pulse using a 90/10 beam splitter.

### Transient Electroluminescence

The Tr‐EL emitted from the QLED was captured using a high‐speed hybrid detector (HPM‐100‐50, Hamamatsu) with a time resolution of 25 ns, and analyzed with a time‐correlated single‐photon counting (TCPSC) module (SPC‐150, Becker & Hickl GmbH).

## Conflict of Interest

The authors declare no conflict of interest.

## Author Contributions

X.Y. and X.Z. contributed equally to this work. B.W., T.W., and S.J. conceived the idea and organized the study; X.Y. performed E‐TA measurements; X.Z. and Y.J. fabricated the QLED devices; X.Y., B.W., and T.W. contributed to the data analysis; B.W., T.W., Y.J., and S.J. wrote the paper with the discussion with all authors.

## Supporting information



Supporting Information

## Data Availability

The data that support the findings of this study are available from the corresponding author upon reasonable request.
