# Supplementary material for: Quantifying Efficiency Roll‐Off Factors in Quantum‐Dot Light‐Emitting Diodes
Source: Adv Sci (Weinh). 2024 Oct 23;11(46):2410041. doi: 10.1002/advs.202410041 (PMC11633546; doi:10.1002/advs.202410041)
Supplement: Supplementary file 1 — Supporting Information [file ADVS-11-2410041-s001.docx]

**Supporting Information**

**Quantifying Efficiency Roll-Off Factors in Quantum-Dot Light-Emitting Diodes**

**The Supporting Information contains the following content:**

**Figure S1:** Electroluminescence (EL) spectra of the QLED.

**Figures S2 – S5:** Demonstration that the formation of the leakage signal requires electrons in the ETL.

**Figure S6:** Temperature of the QD layer at various current densities.

**Figure S7:** E-TA spectra of the QLED as a function of delay time or pump voltage.

**Figures S8 – S11:** Decomposition of the E-TA spectra into bleach, Stark effect, and leakage signals, and the dynamics of each signal.

**Figure S12:** Quantum yield of the QDs as a function of Stark effect signal intensity.

**Figure S13:** Calculation of the number of electrons per QD (*N_e_*) from the intensity of the bleach signal.

**Figure S14:** Measurement of the trion efficiency of QDs.

**Figure S15:** Calculation of the fraction of QDs containing 1 or 2 electrons as a function of *N_e_*.

**Figure S16:** Contribution of each factor toward efficiency roll-off in the case that additional electron charging energy is lower.

**Figure S17:** Correlation between the amplitude of the leakage signal and both the external quantum efficiency (*η*_EQE_) and *N_e_* across different QLEDs.

**Figure S18 – S19:** Current density-voltage-luminance (L-J-V) curves and comparison of leakage signal and EQE for various QLEDs.

**Figure S20 – S21:** Correlation between roll-off and Stark/bleach signals.

**Figure S22:** Verification of the correlation between roll-off and leakage in blue QLEDs.


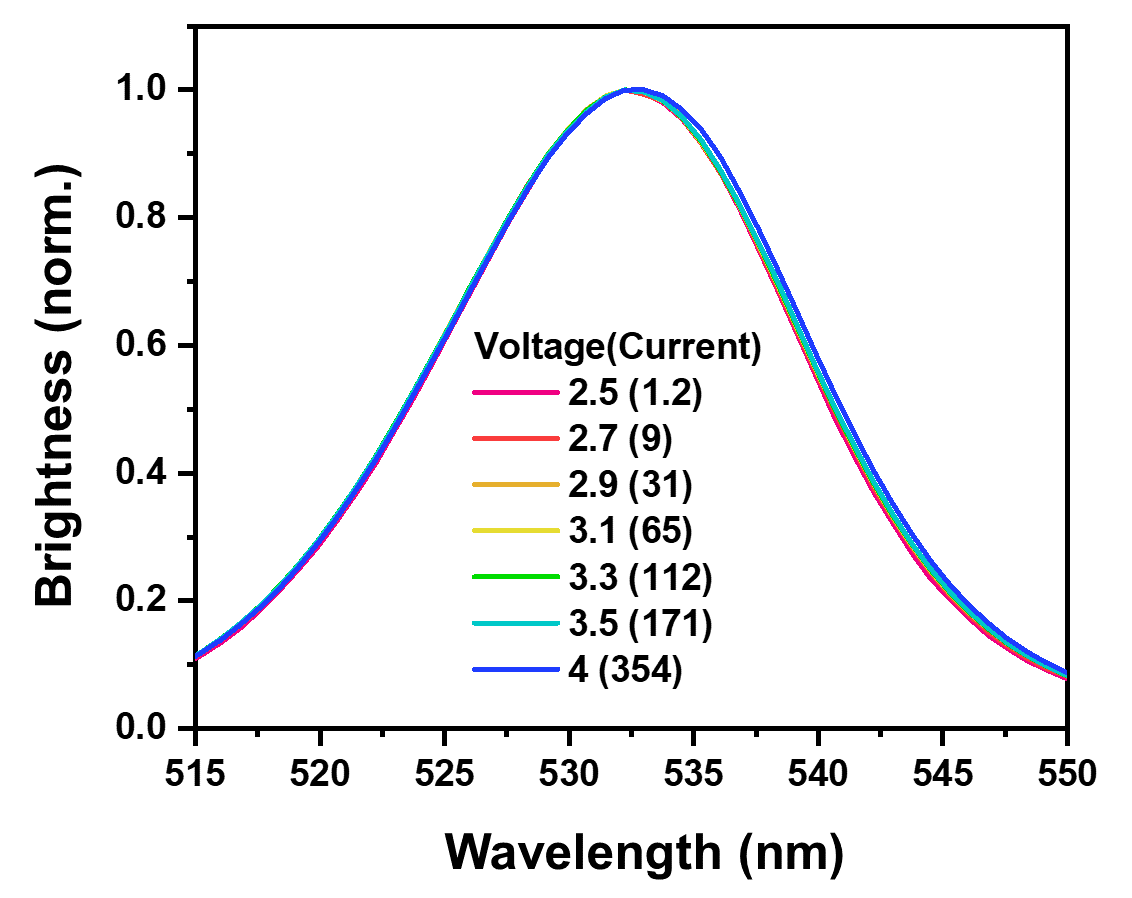


**Figure S1.** Normalized EL emission spectra of the green QLED. The corresponding voltage (in V) and current density (in mA cm^-2^) are in the legends.

**Exploring the origin of the leakage signal**

In this section, we demonstrate that the formation of the leakage signal requires the presence of electrons in the HTL, which in QLEDs can only originate from electrons leaking from the QDs. We fabricated three types of customized devices, with their structures shown in Figure S2(a). The first type (labeled ‘electron and hole’) injects both electrons and holes into the HTL material, PF8Cz. The second type (labeled ‘hole-only’) injects only holes into PF8Cz. The third type (labeled ‘electron-only’) injects only electrons into PF8Cz. As shown in Figure S2(b), a broadband signal similar to the leakage signal in QLEDs is observed only in the ‘electron and hole’ devices. Figure S2(c) compares the E-TA spectra of ‘electron and hole’ device with the actual QLED, revealing an identical bandshape between 550 nm to 750 nm range. Therefore, we conclude that the leakage signal is the same as the absorption signal observed in the HTL, which forms only when both holes and electrons are injected into the HTL.


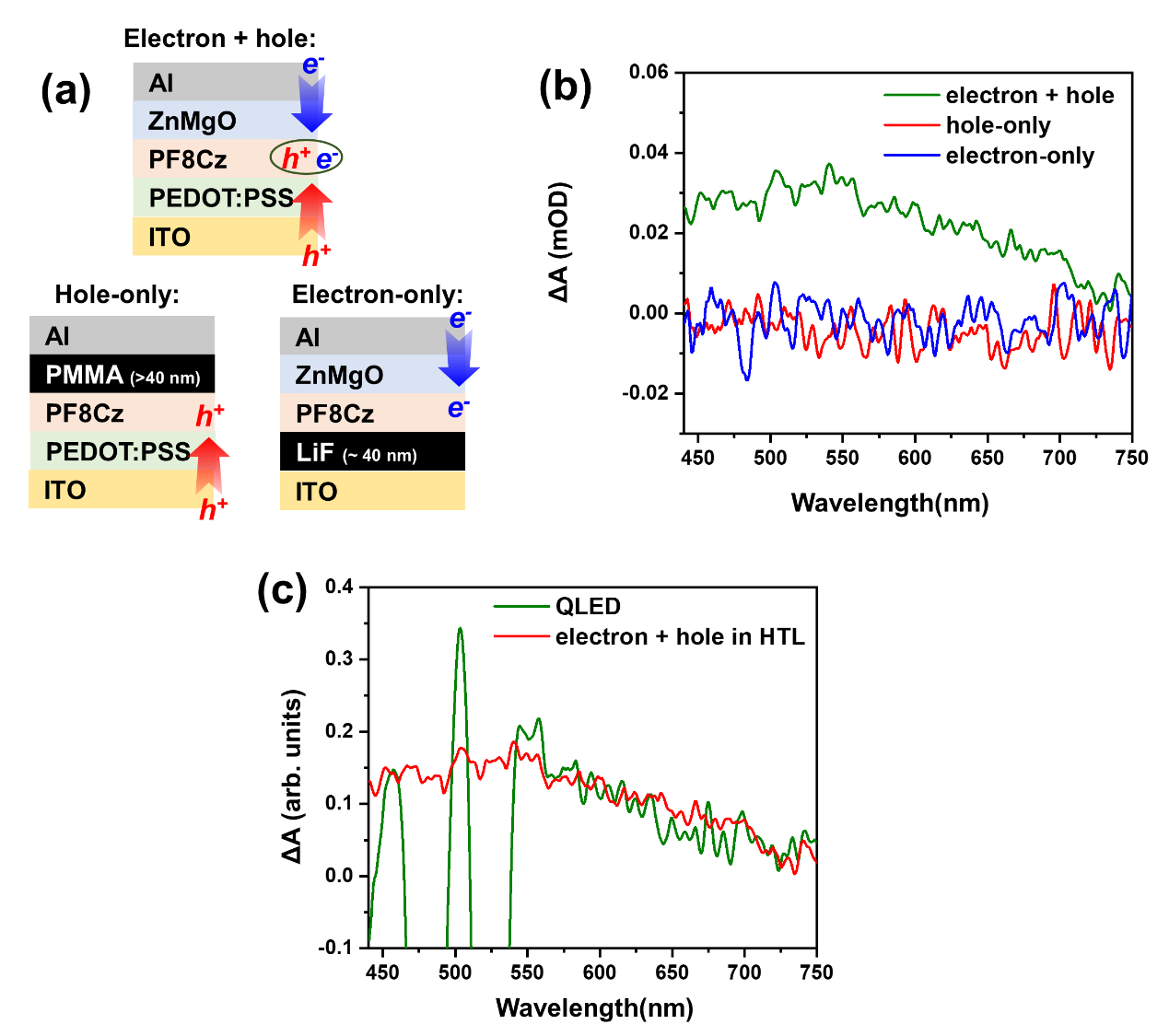


**Figure S2. ETA measurements of three customized devices that injects holes, electrons or electrons + holes into PF8Cz, the HTL material.** (a) The structures of three different customized devices where the HTL is injected with electrons + holes, only holes, or only electrons. (b) E-TA spectra of each device type under a pump voltage of 5V. A broad-band absorbance, identical in bandshape to the leakage signal, is observed only in the device where the HTL contains excitons. (c) Comparison of the E-TA spectrum of the ‘electron + hole’ device with that of an actual QLED.

To further confirm the relationship between leakage signal and excitons in the HTL, we compare its buildup dynamics with that of HTL emission in QLED. This comparison is conducted on QLEDs with TFB as the HTL, since the HTL emission is not observable in QLEDs that use PF8Cz as HTL (as shown in Figure S1). The time-resolved electroluminescence (Tr-EL) of the HTL emission is collected using the same method as that of the QD layer. The difference is that QD emission is collected using a 545±75 nm bandpass filter, while HTL emission is collected using a 430±10 filter. In Figures S3(c)–(f), we compare the rising dynamics of HTL emission and the leakage signal in 2 QLEDs under different pump voltages. The synchronized rising dynamics indicate that the leakage signal and HTL emission have a similar origin, with the latter being caused by excitons in the HTL. However, the leakage signal exhibits an extraordinarily long lifetime compared to the short decay of HTL emission. This suggests that the leakage signal is the triplet state absorption instead of absorption from singlet excitons.


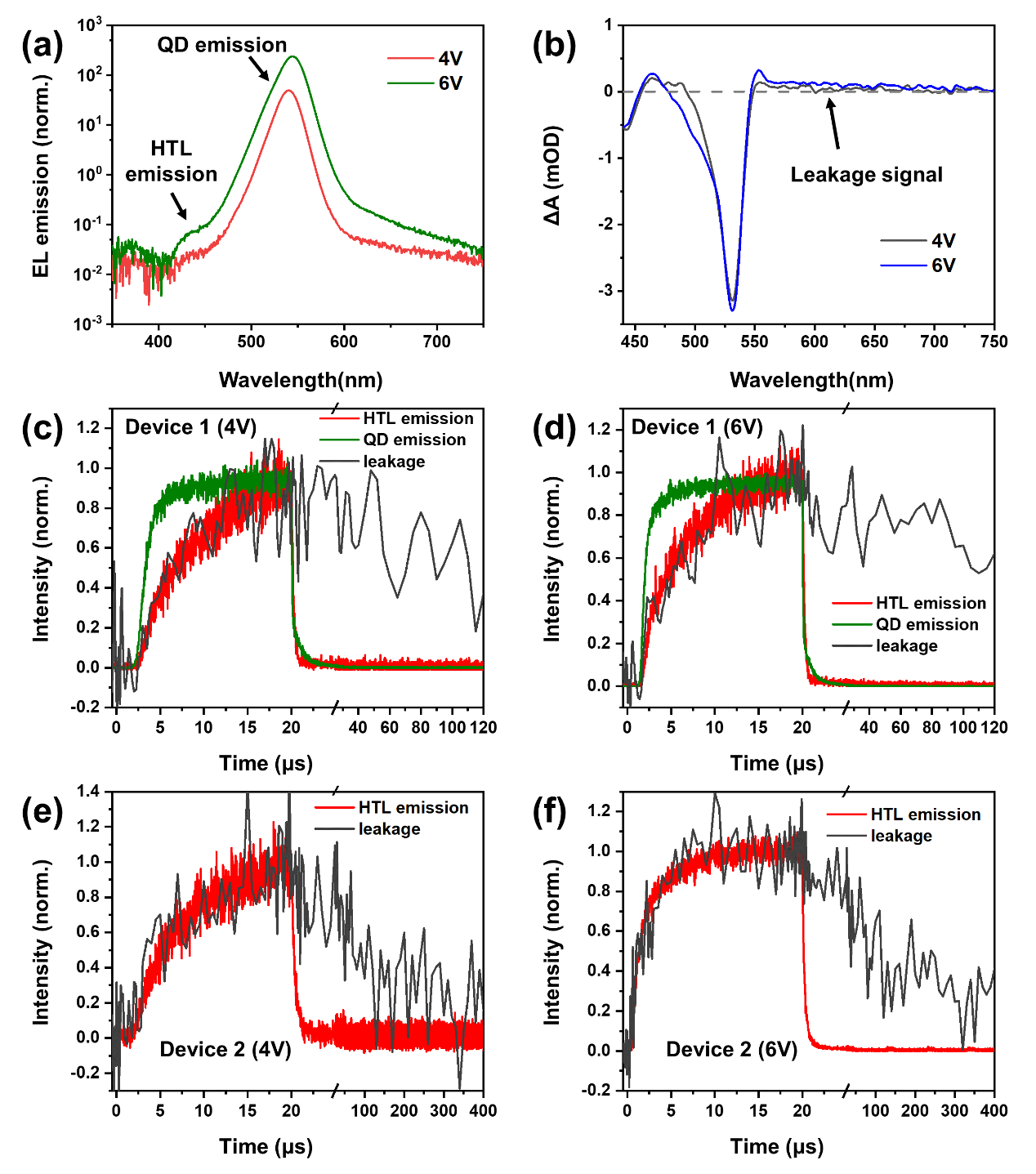


**Figure S3. Comparison of the dynamics of the leakage signal and HTL emission.** The experiment is conducted on a QLED using TFB as the HTL, since HTL emission is not observed in QLEDs using PF8Cz as the HTL. (a) EL emission spectrum of the QLED, indicating the HTL emission and QD emission. (b) E-TA spectra of the QLED, showing the leakage signal. (c)-(f) Comparison of the dynamics of HTL emission and leakage signals in two different QLEDs (with the same structure) at different pump voltages. In (c) and (d), the dynamics of QD emission are also presented.

To further identify the leakage signal as originating from a triplet excited state absorption of the HTL, we take the long-time optically pumped transient absorption spectrum of PF8Cz (the HTL material). The optical transient absorption spectrum and dynamics of a PF8Cz film are shown in Figures S4(a) and S4(b), respectively. The absorbance signal takes 3 ns to build up, consistent with the intersystem crossing time scale of excitons transitioning from singlet to triplet states in typical organic polymers.^[1-3]^The signal then persists for over 400 µs before disappearing, indicating a very long lifetime and confirming its identification as a triplet state. This observation explains the data in Figures 3(c)–(f): the signal's rapid buildup (< 3 ns) allows it to synchronize with the HTL emission, and its very long lifetime corresponds to the slow decay of the leakage signal.


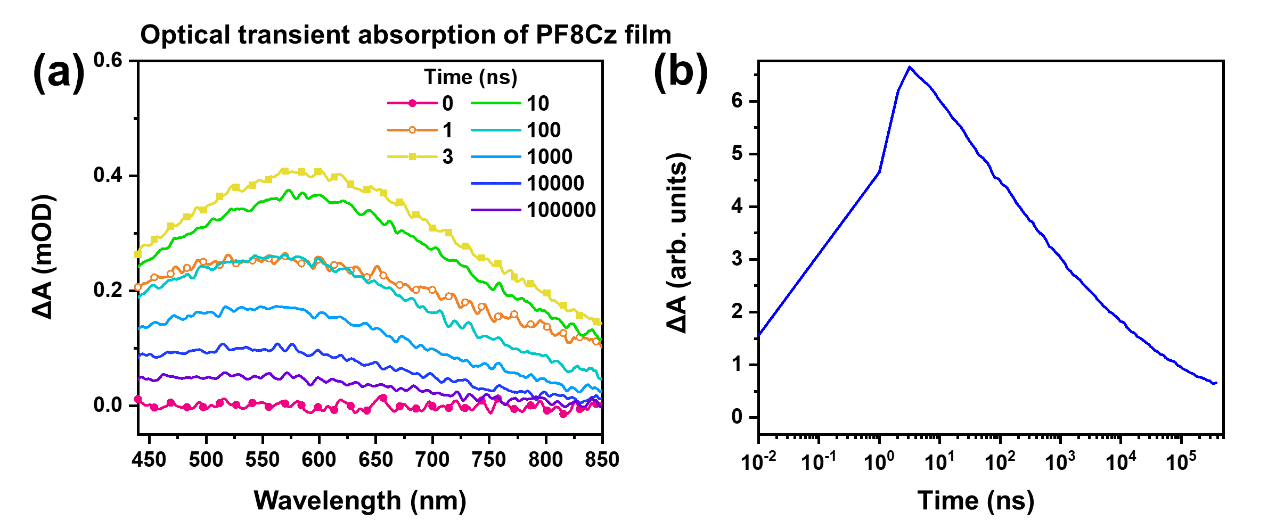


**Figure S4. Optical transient absorption measurements of a PF8Cz film.** (a) Transient absorption spectrum of the PF8Cz film at different delay times. Transient absorption measurements with delay times up to 400 µs are achieved using an electrical time delay generator, the same as that used in the E-TA measurement, instead of the traditional mechanical delay line. (b) Dynamics of the transient absorption signal (integrated between 500 nm and 700 nm), which reaches its maximum at 3 ns and begins a long decay lasting from 3 ns to 400 µs.

We also measure the optical transient absorption spectrum of a film of QDs (Figure S5) and observed no broadband absorption, further confirming that the leakage signal originates from the HTL. Based on the analysis above, we believe the leakage signal should be attributed to the triplet state absorption of PF8Cz, and we are confident to conclude that the formation of this signal requires the presence of electrons in the HTL, making it a measure of electron leakage into the HTL from the QDs.


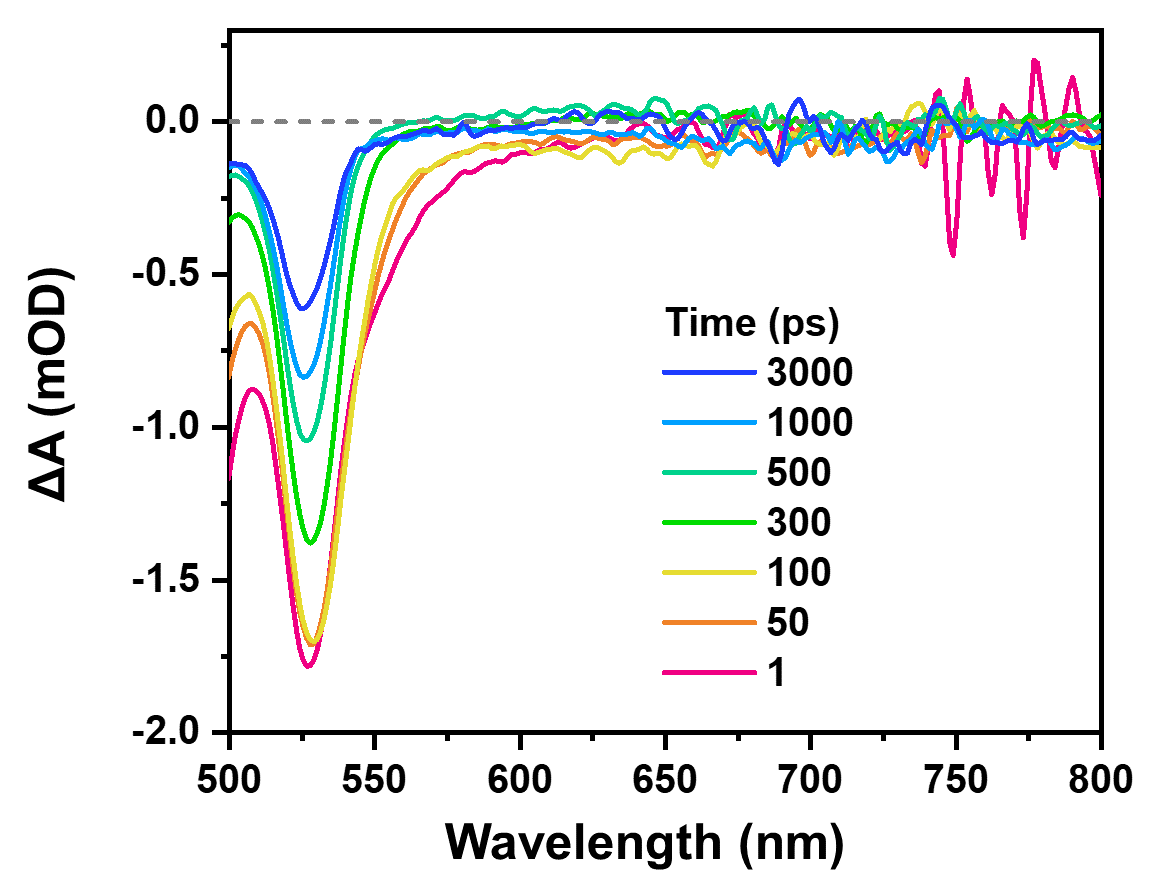


**Figure S5.** Optically pumped transient absorption spectrum of a QD film. No broadband absorption is observed between 600 nm and 700 nm.


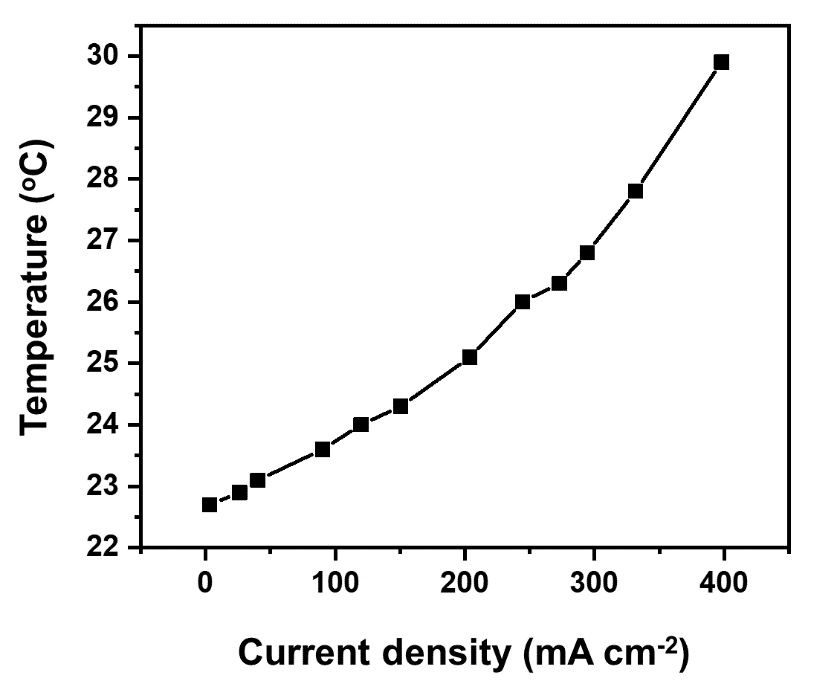


**Figure S6. Temperature of QDs during operation.** The temperature of the QLED during operation was measured using a micro thermocouple attached to the emission area. The observed temperature change is minimal, indicating that Joule heat-induced quenching is not significant in the QLED under study.


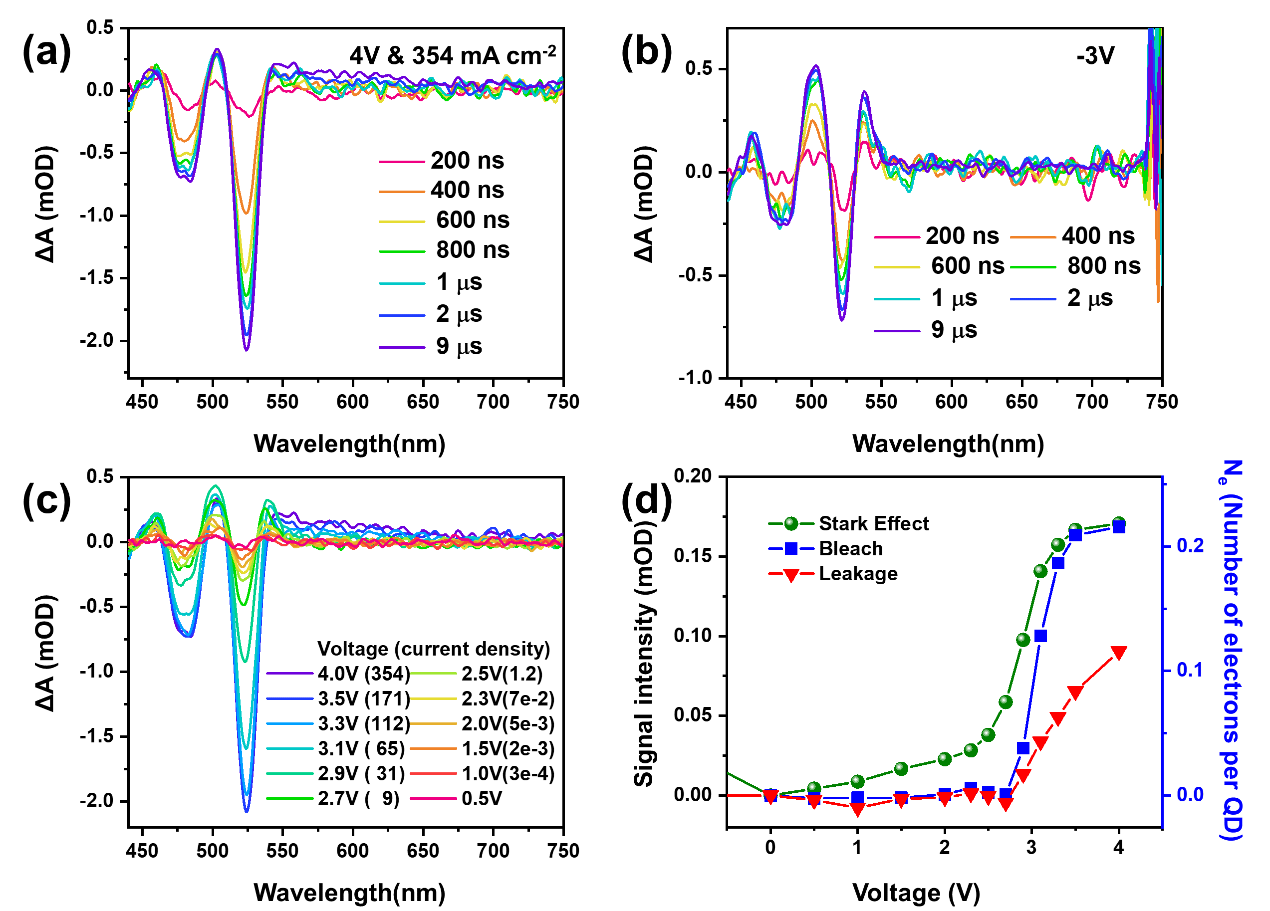


**Figure S7.** (a) and (b) The time-resolved E-TA spectra of the green QLED at pump voltages of 4V forward voltage and 3V backward voltage (denoted as -3V). (c) Equivalent of Figure 3 (c) in the main text, where both pump voltage and current is in the legend. (d) Equivalent of Figure 3 (d) in the main text, where x-axis is pump voltage instead of current density.

**Decomposing the E-TA spectra into bleach, Stark effect, and bleach signals**

We performed spectral decomposition using a global fit based on the least-squares fitting method. To apply this approach, we measured the E-TA spectra, ΔA_E-TA_(*λ, T_d_, V*), as a function of wavelength (*λ*) at various delay times (T_d_) and voltages (*V*), including forward and reverse biases. The E-TA spectrum is modeled as a linear combination of the following components: Stark effect signal (*S(λ)*), bleach signal (*B(λ)*), and leakage signal (*L(λ)*).

$$\begin{aligned} {\Delta A}_{E-TA}\left( \lambda, T_{d},V \right)=C_{1}(T_{d},V)*S\left( \lambda\right)+C_{2}(T_{d},V)*B\left( \lambda\right)+C_{3}(T_{d},V)*L\left( \lambda\right)\#\left( AUTONUM \backslash* Arabic \right) \end{aligned}$$

where, *C*_1_, *C*_2_, and *C*_3_ are functions of T_d_ and *V*, while the bandshapes of *S(λ)*), (*B(λ)*), and (*L(λ)*) remain unchanged when T_d_ or *V* are different.

The shape of the bleach signal, *B(λ)*, is simulated using a multi-Gaussian equation:

$\begin{aligned} B\left( \lambda\right)=-\sum_{n} \left( {a_{n}\cdot e}^{-\frac{\left( \lambda-c_{n} \right)^{2}}{2{\omega_{n}}^{2}}} \right)\#\left( AUTONUM \backslash* Arabic \right) \end{aligned}$

where *a_n_*, *c_n_*, and *ω_n_* are float parameters being optimized during the global fits. The shape of the Stark effect signal, *S(λ)*, is modeled as a linear combination of the first-order and second-order derivatives of multi-Gaussian peaks. These derivatives represent the shift (first derivative) and broadening (second derivative) of the QD absorption peak under the influence of an electric field.^[4-5]^:

$$\begin{aligned} G\left( \lambda\right)=\sum_{n} \left( {a_{n}\cdot e}^{-\frac{\left( \lambda-c_{n} \right)^{2}}{2{\omega_{n}}^{2}}} \right)\#\left( AUTONUM \backslash* Arabic \right) \end{aligned}$$

$$\begin{aligned} S\left( \lambda\right)=G^{'}\left( \lambda\right)+k\cdot G^{''}\left( \lambda\right)\#\left( AUTONUM \backslash* Arabic \right) \end{aligned}$$

where *a_n_*, *c_n_*, and *ω_n_* are also float parameters being optimized during the global fits. *G'(λ)* and *G''(λ)* represent the first and second derivatives of *G(λ)*, respectively. The shape of the leakage signal, *L(λ)*, is fixed during the global fits and constrained to the experimentally measured triplet-excited absorption bandshape of PF8Cz as shown in Figure S4(a). We determine the values of *C*_1_, *C*_2_, and *C*_3_ (in Equation (1)) and the bandshapes of *S(λ)* and *B(λ)* through global fitting, minimizing the difference between the experimentally measured ΔA_E-TA_(*λ, T_d_, V*) and the values calculated by Equation (1) using a least-squares method.

During the global fits, the bandshape of each signal type converges to a reasonable form due to the dominance of different signal types under different conditions. For example, under reverse biases, only the Stark signal is present, which facilitates the convergence of *S(λ)* since both the bleach and leakage signals are absent. Conversely, the leakage signal appears only under high voltage and long T_d_ conditions. After the global fits, we can extract the bleach, Stark, and leakage components

$$\begin{aligned} {\Delta A}_{\mathrm{Stark}}\left( \lambda, T_{d},V \right)= C_{1}(T_{d},V)*S\left( \lambda\right)\#\left( AUTONUM \backslash* Arabic \right) \end{aligned}$$

$$\begin{aligned} {\Delta A}_{\mathrm{bleach}}\left( \lambda, T_{d},V \right)= C_{2}(T_{d},V)*B\left( \lambda\right)\#\left( AUTONUM \backslash* Arabic \right) \end{aligned}$$

$$\begin{aligned} {\Delta A}_{\mathrm{leakage}}\left( \lambda, T_{d},V \right)= C_{3}(T_{d},V)*L\left( \lambda\right)\#\left( AUTONUM \backslash* Arabic \right) \end{aligned}$$

In Figure 2(b) in the main text, we present ΔA_E-TA_(λ), ΔA_bleach_(λ), ΔA_Stark_(λ), and ΔA_leakage_(λ) for a voltage of 4V (corresponding to a current density of 354 mA cm^-2^), averaged for T_d_ > 8 μs, where the bandshape has converged. Figure S8 shows the corresponding data for different T_d_, while Figure S10 presents the decomposition for various voltages.

Finally, the signal intensities in Figure S3(d) in the main text are calculated by averaging the absolute values of the corresponding signals over a wavelength range from 440 nm to 750 nm and for T_d_ > 8 μs:

$$\begin{aligned} {\Delta A}_{Stark/Bleach/Leakage}\left( V \right)= {|\Delta A}_{Stark/Bleach/Leakage}\left( 440 nm<\lambda< 750 nm, T_{d} > 8 \mu s,V \right)|\#\left( AUTONUM \backslash* Arabic \right) \end{aligned}$$

Using the J-V curve, the signal intensities are converted into a function of current density as ΔA_Stark/Bleach/Leakage_(ρ).


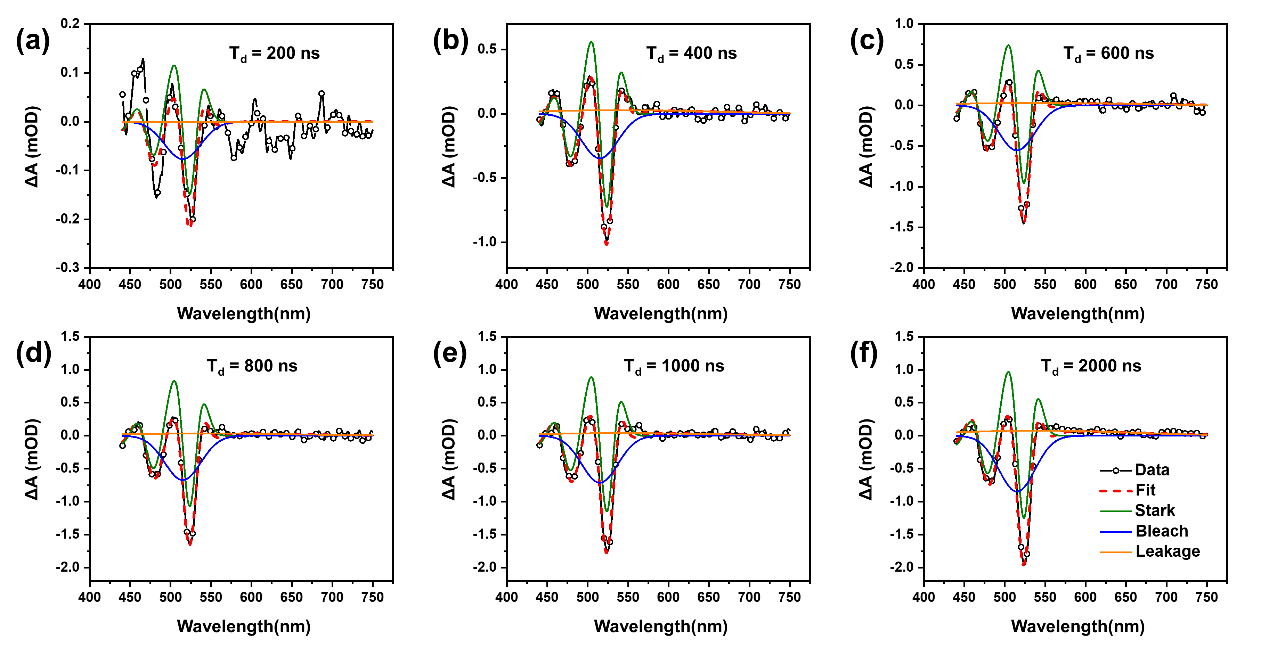


**Figure S8.** The decomposition of E-TA spectra at different T_d_ in Figure S3 into the Stark, bleach, and leakage signals.


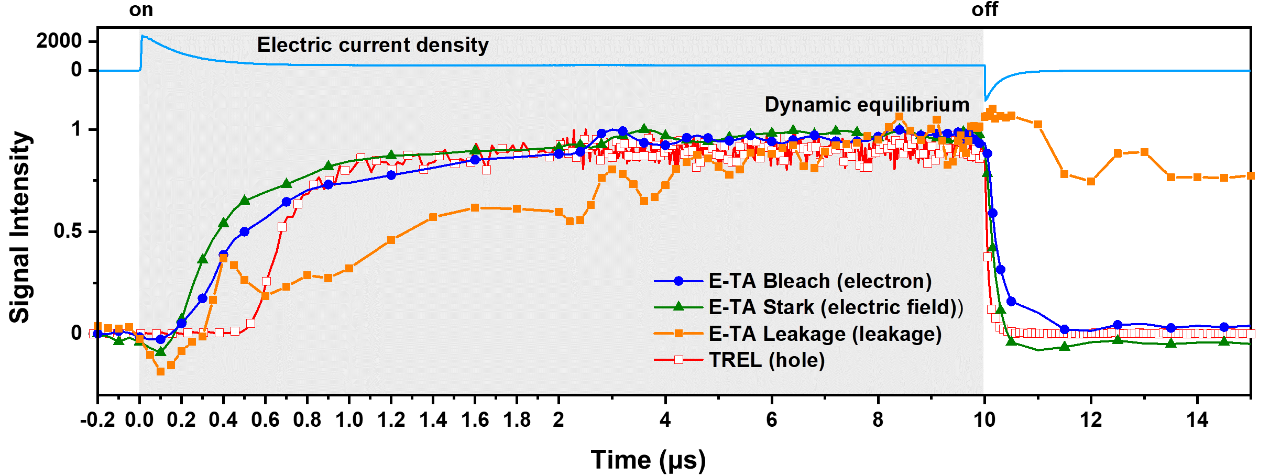


**Figure S9.** Comparison of the dynamics of the bleach, Stark, and leakage signals at 4V in a single graph. Time-resolved electroluminescence (Tr-EL) and transient current under the same voltage excitation pulses are also presented. Tr-EL data is collected using a high-speed hybrid detector (HPM-100-50, Hamamatsu) and analyzed via a time-correlated single-photon counting (TCPSC) module (SPC-150, Becker & Hickl GmbH) operating under a multi-channel scaler (MCS) mode with a 25 ns time resolution. Transient current measurements are acquired using a current probe (Tektronix TCP0030A, 120MHz) and an oscilloscope (Tektronix MSO44, 500MHz).


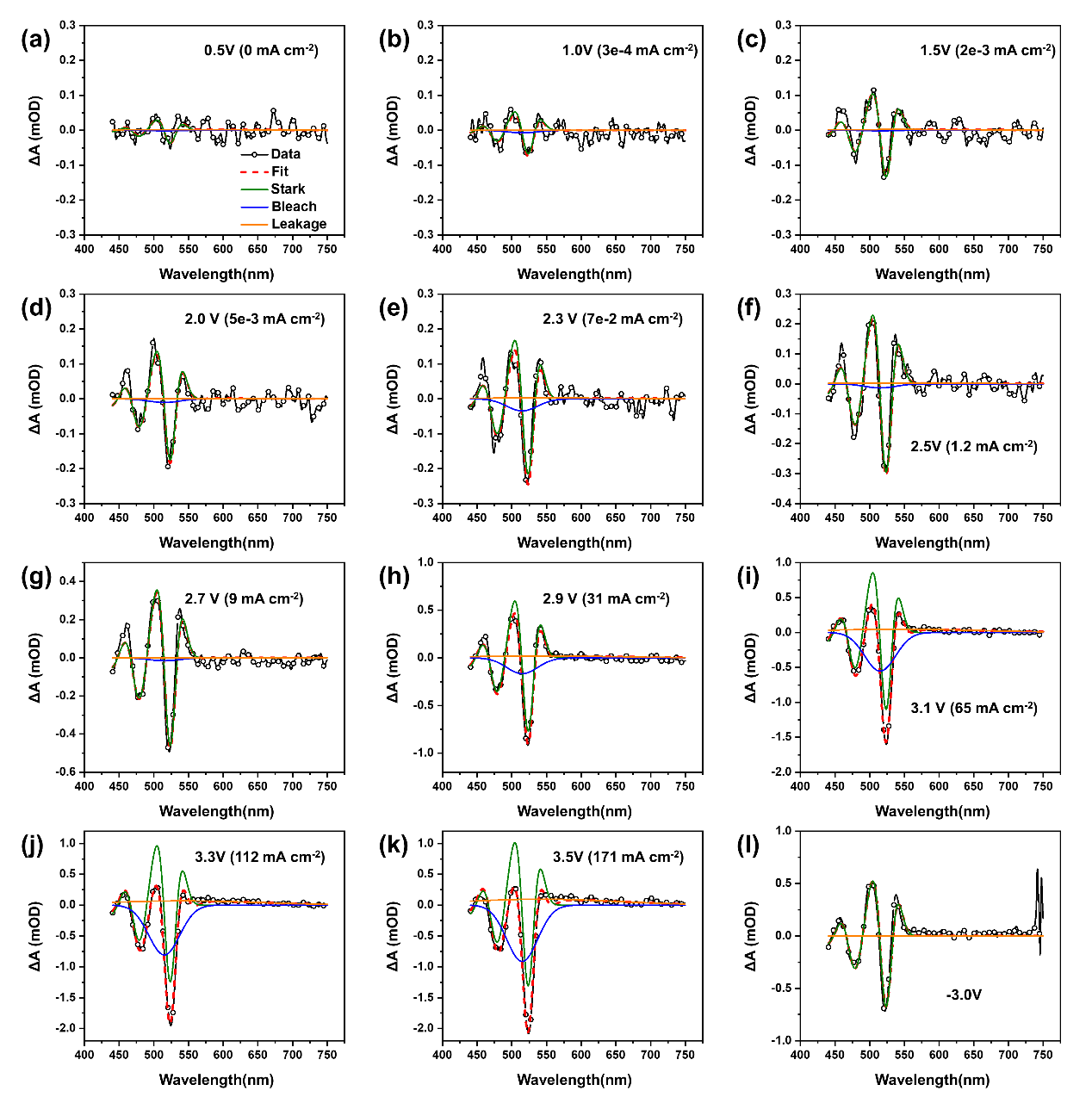


**Figure S10.** Decomposing the E-TA spectra at various pump voltages into the Stark, bleach, and leakage signals, with the pump voltage and corresponding current indicated in each plot.


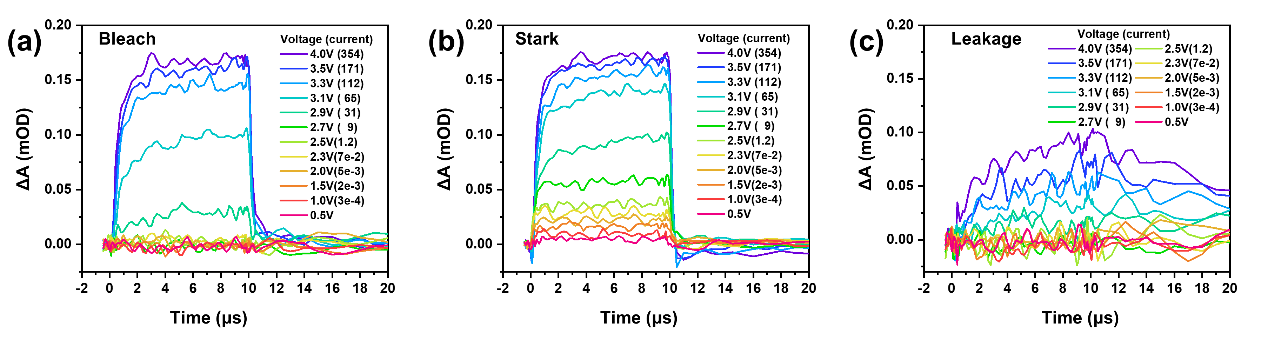


**Figure S11.** The time-dependent intensity of bleach, Stark and leakage signals at different pump voltages/current densities.

**Evaluate the influence of quantum yield of the QDs by electric field.**

Figure S12 shows the process of evaluating the electric field-induced quenching on QDs. The objective is to determine the relative quantum yield of the QDs (*η*_Stark_) as a function of Stark effect signal intensity that presented in Figure 4(a) in the main text.

To achieve this, we fabricate customized devices as shown in Figure S12, where both sides of the QD layer are blocked for carrier injection by insulator PMMA polymer. The E-TA spectra of one such device under different pump voltages are presented in Figure S12 (a). Integrating the spectra in (a) along the wavelength axis yields zero, confirming that only the Stark effect signal is observed, with no bleach signal. This indicates that no electrons enter the QD layer in this customized device. At identical voltages, we measure its photoluminescence (PL) spectra and PL lifetime using 450 nm laser pulse excitation. The PL spectra and decay are displayed in Figure S12 (b) and (c), respectively. In Figure S12 (d), we plot the computed Stark effect intensity ΔA_Stark_ from spectra in panel (a), averaging the absolute value over the spectral range from 440 nm to 750 nm. Figure S12 (e) displays the relative PL intensity obtained from emission spectra in panel (b), with the value at 0V scaled to 100%. Similarly, Figure S12 (f) displays the PL lifetime obtained from the PL decay in panel (c). Calculating the quenching of QDs from both intensity (e) and lifetime (f) yields comparable results. In panels (d), (e), and (f), measurements from three individual customized devices are presented, which yield similar outcomes. By combining the results from panel (d) and (e), we derive the relative PLQY of the QDs (*η*_Stark_) as a function of Stark effect signal intensity.


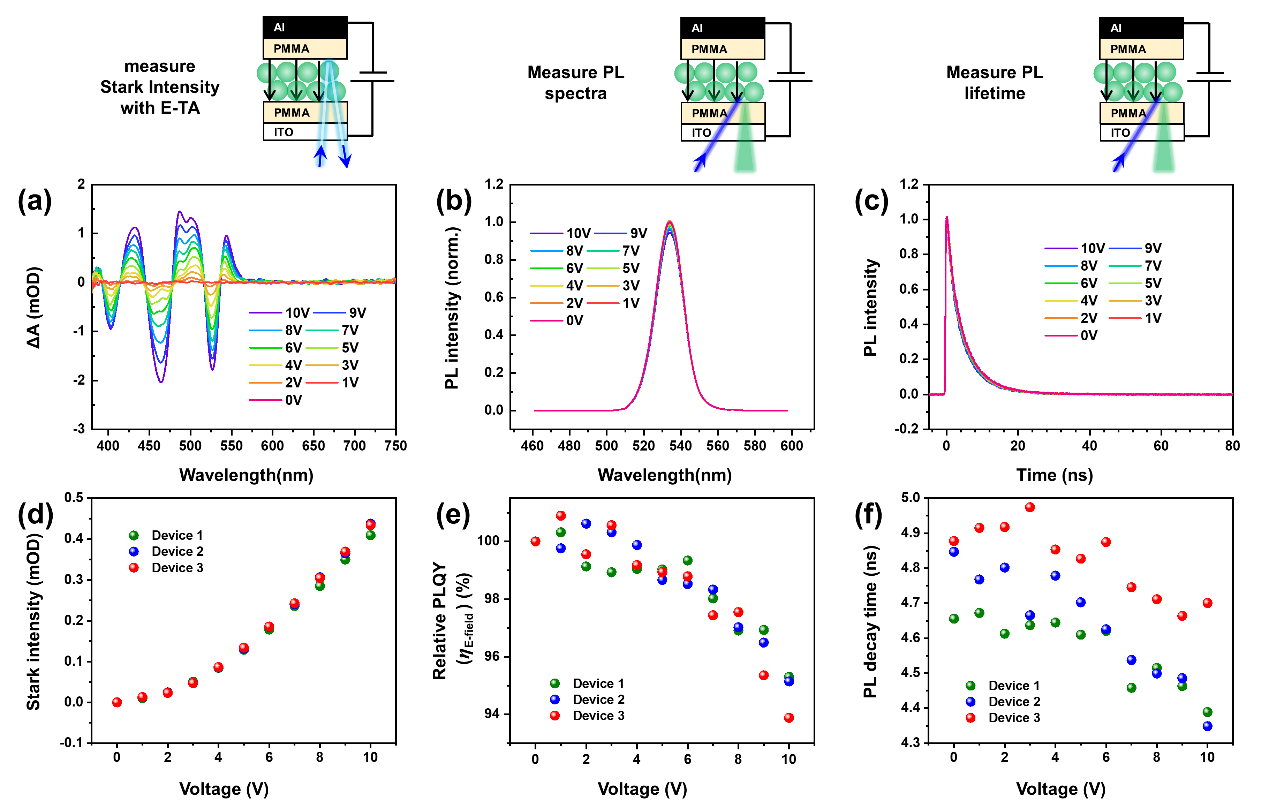


**Figure S12.** (a) E-TA spectra of customized devices where the QD is sandwiched between two insulator PMMA layers at different pumping voltages. (b) PL spectra by optical excitation of these devices with different voltages applied. (c) Time-resolved PL by optical excitation of these devices under different applied voltages. (d) Stark effect intensity as a function of applied voltage, with three colors of dots representing three individual devices obtained from (a). (e) Relative PLQY of the three devices, calculated from spectra in (b). (f) PL lifetime of the devices obtained from (c).

**Calculation of *N*_e_ from bleach signal**

According to Lambert-Beer's law, the number of electrons per QD, *N_e_*, is proportional to the bleach signal intensity, with the relationship given by *N_e_* = *α* × ΔA_bleach_. The coefficient *α* is determined from optically pumped transient absorption experiments on the QLED. When the QLED is pumped with light pulses, a bleach signal arises from the quenching of the 1S [1S_3/2_(h) – 1S(e)] transition due to the occupation of electrons of 1S orbital and holes on 1S_3/2_(h) orbital. Since optical transient absorption bleach signal has the same nature as the E-TA bleach signal, their relationship between *N_e_* and ΔA_bleach_ is also the same. As the intensity of the optical pump light increases, the intensity of bleach signal also increases until it reaches saturation. The intensity of bleach signal at saturation point, corresponding to Ne =2, is used to determine the coefficient *α*, yielding a value of 1280.

Here is a detailed description of the process used to determine the correlation:

When the QLED is pumped with optical pulses, the number of excitons on per QDs follows a Poisson distribution,^[6]^

$$\begin{aligned} P_{<N>}(n)=\frac{e^{-<N>}\cdot<{N>}^{n}}{n!}\#\left( AUTONUM \backslash* Arabic \right) \end{aligned}$$

where <N> represents the average number of excitons per QDs, calculated as the product of the excitation light photon flux (proportional to the excitation light power) and the absorption cross-section of the QDs.

In Figure S13 (a), TA spectra at 100 ps under different pump powers are plotted. The bleach signal of 1S [1S_3/2_(h) – 1S(e)] transition near the 528 nm center is observed, which is caused by the filling of the electron in the 1S(e) orbitals. The CdSe QDs 1S(e) level is 2-fold degenerate, after pulsed optical excitation and thermal relaxation, it can hold 0, 1, or 2 electrons, which correspond the no bleach, half bleach and complete bleach. According to the Poisson distribution, the probabilities for these states are $P_{0}=P_{<N>}(0)=e^{-<N>}$, $P_{1}=P_{<N>}\left( 1 \right)=e^{-<N>}\cdot<N>$, and $P_{2}={1-P_{0}-P}_{1}$. Since *N_e_* is calculated based on the 1S transition bleach signal intensity, the *N_e_* is equivalent to the average number of electrons in the QDs 1S(e) level and can be expressed as,^[7]^

$$\begin{aligned} N_{e}=\left( 1-P_{0} \right)e^{-\frac{t}{\tau_{1}}}+(1-P_{0}-P_{1})e^{-\frac{t}{\tau_{2}}}\#\left( AUTONUM \backslash* Arabic \right) \end{aligned}$$

here,$\tau_{1}$ is single exciton decay lifetime and $\tau_{2}$is biexciton decay lifetime. For time points before decay begins, $e^{-t/{\tau_{1}}}$ and $e^{-t/{\tau_{2}}}$ are equal to 1, and *N_e_* can be expressed as,

$$\begin{aligned} N_{e}=2-2e^{-<N>}-e^{-<N>}\cdot<N>\#\left( AUTONUM \backslash* Arabic \right) \end{aligned}$$

When the excitation power is high enough, *N_e_* approach to 2. In Figure S13 (b), TA spectra are plotted as a function of time. The decay dynamics of the 1S transition peak (taken at 528 nm) is shown in panel (c), where a plateau is observed between 10 ps and 100 ps, in which time the relaxing of higher excitations states are filling into 1S transition, but that could not further increase ΔA 1S orbitals have been saturated with electrons or holes. During this plateau, 1S(e) level should be full of electrons, and the ΔA at this time should match the intensity of the bleach signal in the E-TA experiment when *N_e_* = 2.

Furthermore, when sufficient time has passed for biexciton recombination to become negligible, $e^{-t/{\tau_{2}}}$ approaches to 0, and only single exciton decay should be considered in equation 4. At this stage, *N_e_* can be expressed as,

$$\begin{aligned} N_{e}=\left( 1-e^{-<N>} \right)e^{-\frac{t}{\tau_{1}}}\#\left( AUTONUM \backslash* Arabic \right) \end{aligned}$$

When the excitation power is high enough, *N_e_* is equal to $e^{-t/{\tau_{1}}}$. When extrapolating from time t to the time zero based on single-exponential decay, *N_e_* equals to 1. As shown in panel (d), the kinetic curve at 528 nm and 158 µW is plotted, showing a faster decay before 1.5 ns, corresponding to Auger recombination, and a slower single-exponential decay at longer times. Extrapolating the single-exponential decay to time zero, as indicated by the red dashed line, should correspond to where *N_e_* = 1. Panel (e) shows the measured spectra at long time delays as well as their extrapolated spectrum at time zero.

By comparing the bleach signal intensity measured in the E-TA, ΔA_bleach_, with the bleach signal intensities calculated for *N_e_*=1 and *N_e_* =2, we can determine the corresponding *N_e_* value for the bleach signal. This analysis indicates that the coefficient *α*, which correlates ΔA_bleach_ to *N_e_*, is 1280.

The validation of this method is in our previous work.^[7]^ In that study, we assessed the correlation between *N_e_* and ΔA_bleach_ using three different methods. Besides the method described in this work, we also calculated the correlation by measuring the absorption cross-section of the QDs and the QD density. Additionally, we used the E-TA method to achieve saturation of the QD layer: devices with the HTL replaced by a carrier blocking layer were subjected to high voltages in E-TA measurements to saturate them with electrons, resulting in *N_e_* = 1. The correlations between *N_e_* and ΔA_bleach_ obtained from these three methods were found to be consistent.

The femtosecond transient absorption system uses a regenerative amplified Ti:sapphire laser (Coherent, 800 nm, 35 fs, 6 mJ/pulse, and 1 kHz repetition rate) as laser source. The pump beam is generated by a TOPAS Optical Parametric Amplifier (OPA), while the probe white light continuum pulse, delayed by a mechanical time delay, is also generated from the 800 nm source laser by a 2 mm thick sapphire window. The probe light after reflected on the QLED is collected by the Femto-TA 100 spectrometer (Time-Tech Spectra). In the optically pumped transient absorption experiments, the probe pulse goes through the same optical path as in the E-TA spectra, except that it is focused on the QD layer. (Probe beam focusing is unnecessary in an E-TA experiment.) The pump beam overlaps with the probe beam on the QD layer.


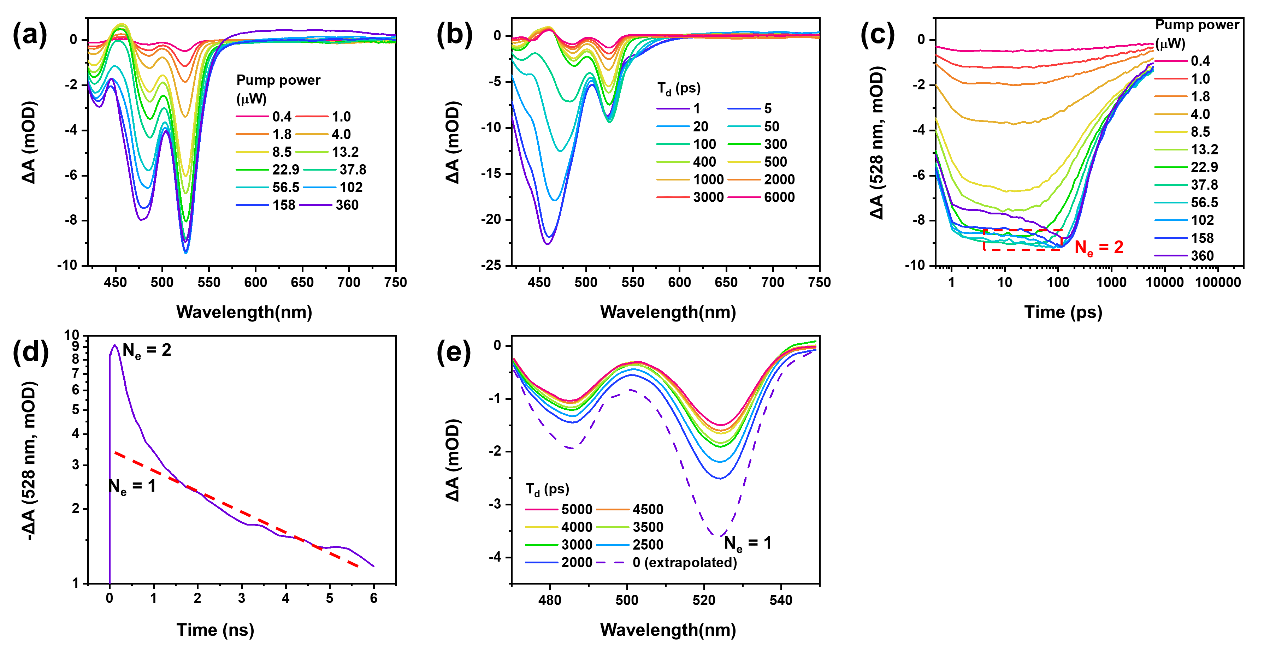


**Figure S13:** (a) Optically pumped transient absorption (TA) spectra of the QLED used in this work at a time delay of 100 ps under different pump energies. (b) TA spectra of QLED under 158 μW pump power at different delay times. (c) TA decay dynamics at 528 nm under different pump powers. (d) TA decay dynamics at 528 nm. The peak absorbance corresponds to N_e_ = 2, and the extrapolation of the later single exponential decay to time zero (red dashed line) represents N_e_ = 1. (e) TA spectra of 158 μW pump power at late times, along with the extrapolated spectra at time zero, corresponding to *N_e_* = 1.

**Measurement the trion efficiency of QDs:**

To assess the relative efficiency of negatively charged trions, we prepare a toluene solution containing quantum dots (QD) and negatively charged quantum dots (QD^-^) by mixing QDs with lithium triethylborohydride, an electron donor, under continuous light excitation from a 365 nm UV lamp. Both the solution of pure QDs and the QD-QD^-^ mixture are excited using 450 nm laser pulses, and their PL decay dynamics are represented by the red and black curves in Figure S14 (b), respectively. These two curves are aligned to synchronize the long-time single exponential decay, representing the decay of neutral excitons (X). By subtracting the decay curve of X from the red curve, we isolate the PL decay of X^-^ (blue curve), from which we can determine its lifetime.


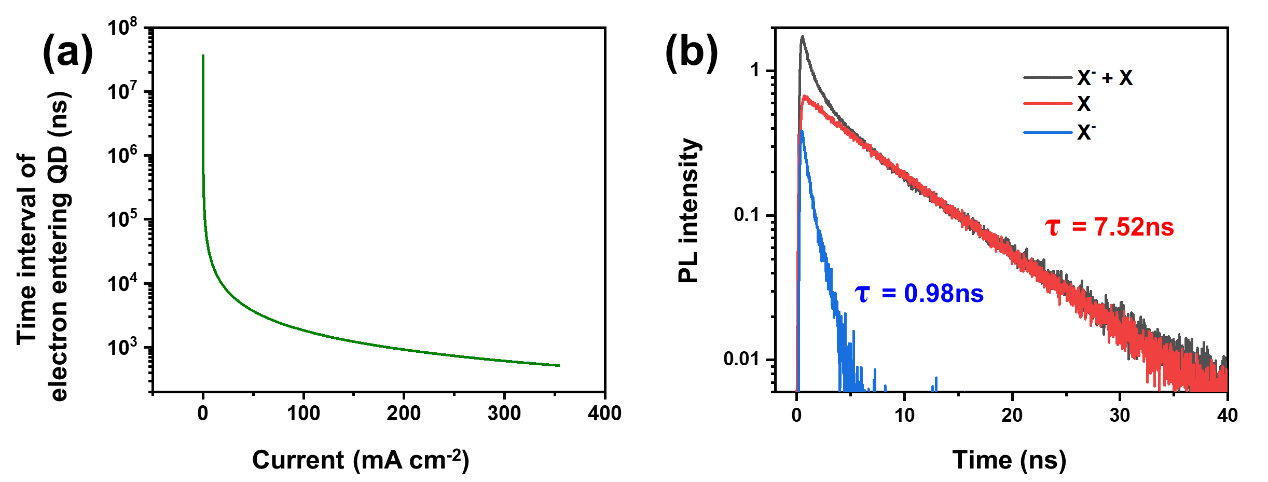


**Figure S14.** (a) Averaged time interval between electron injections into each QD computed based on the current density. (b) Measurement of the lifetime of neutral excitons (X) and negative trions (X^-^) in the QDs.

**Calculation of P(1) and P(2)**

The value of P(2) is simulated using a Monte Carlo method following this algorithm: Initially, the QD layer is empty of electrons. Electrons are then added into the QDs one by one until the total occupation number reaches 𝑁_𝑒_​. The probability that an electron enters an empty QD is greater than the probability of entering a QD that is already occupied by an electron by a factor of 1/𝑍, where 𝑍 is the partition function given by Z=exp(-E_c_​/*k*_B_​T). Here, 𝑘_𝐵​_ is the Boltzmann constant, and 𝑇 is the temperature. Given the relatively low averaged *N_e_* value and the substantial energy barrier between the 1S and 1P orbitals of the QDs, we determine that each QD can only accommodate 0, 1, or 2 electrons, with a third electron unable to enter.

The simulation result is displayed in Figure S15 , where the value of P(2) is plotted as a function of *N_e_* for different E_c_. When E_c_ = 0, the distribution of electrons among the QDs is close to a Poisson distribution (except that the situation that more than 2 electrons in one QD is disallowed).

In our case, where the radius of the QDs is 1.5 nm, E_c_ = 150 meV according to literature value of QDs of the same material and radius.^[8-9]^ Calculating P(2) with E_c_ = 150 meV yields a very small fraction of QD^2-^, indicating that the efficiency loss caused by Auger recombination is minimal.


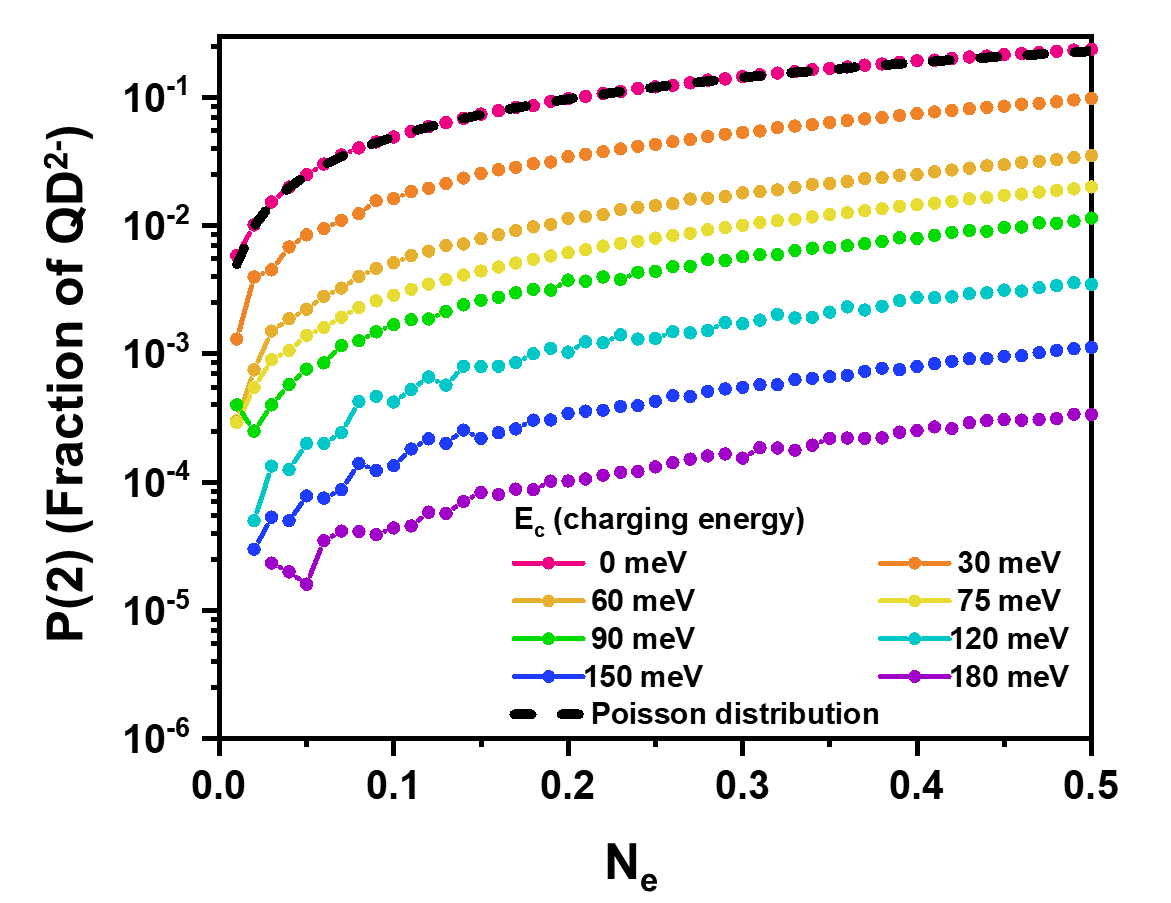


**Figure S15.** P(2) (fraction of QD^2-^ within all negatively charged QDs) as a function of *N_e_* determined through a Monte Carlo simulation. E_c_ is the additional electron charging energy into a QD already occupied by an electron. The black dashed line represents the results of a Poisson distribution.

In Figure S16 (a), we compare *η*_Auger_ in the QLED as a function of current density computed with E_c_ = 0 and E_c_ = 150 meV, respectively, after incorporating the measured *N_e_* at different current densities. In Figure S16 (b), we recalculate the contribution of each roll-off factor while considering E_c_ = 0. This shows the theoretical maximum contribution of Auger recombination, which is still significantly smaller than the contribution of leakage at 345 mA cm^-2^.


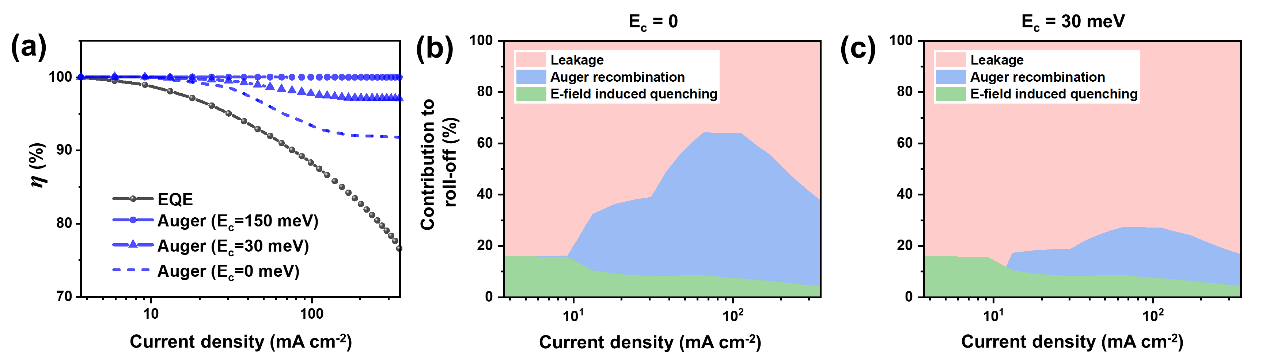


**Figure S16.** Comparison the relative residual efficiency after Auger recombination (η_Auger_) computed with E_c_ = 0, 30, and 150 meV. (b) The contribution of each factor while considering E_c_ = 0, where the electron distribution within the QDs obeys Poisson distribution. (c) The contribution of each factor while considering E_c_ = 30 meV.


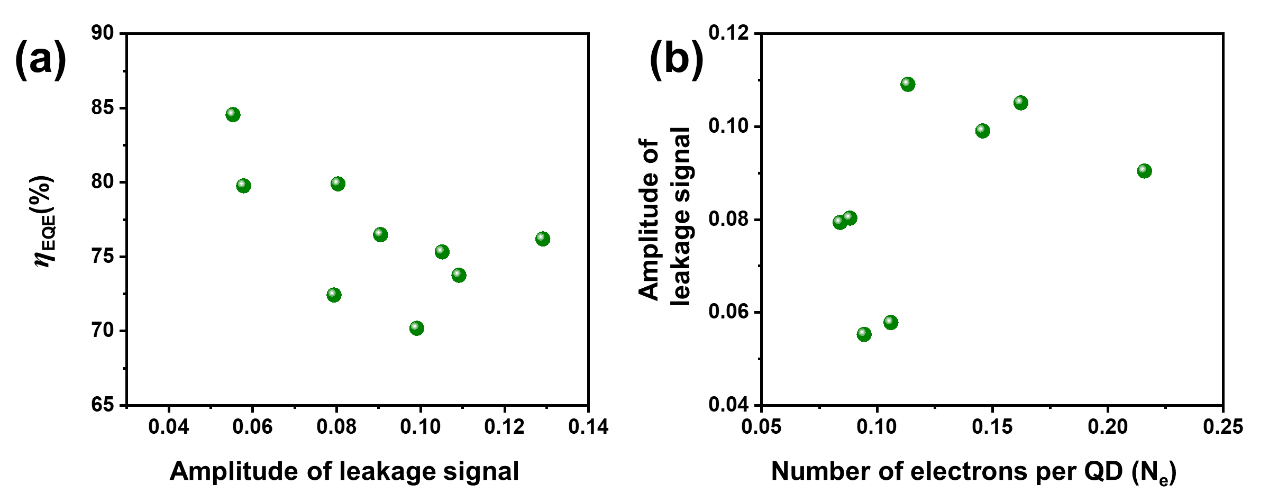


**Figure S17.** (a) The correlation between the amplitude of the leakage signal and *η*_EQE_, including data from 9 distinct QLEDs all operating at a voltage of 4 V. Each data point represents an individual QLED. (b) The correlation between *N_e_* and the amplitude of the leakage signal, indicating that as more electrons accumulate in the QDs, electron leakage becomes more probable.


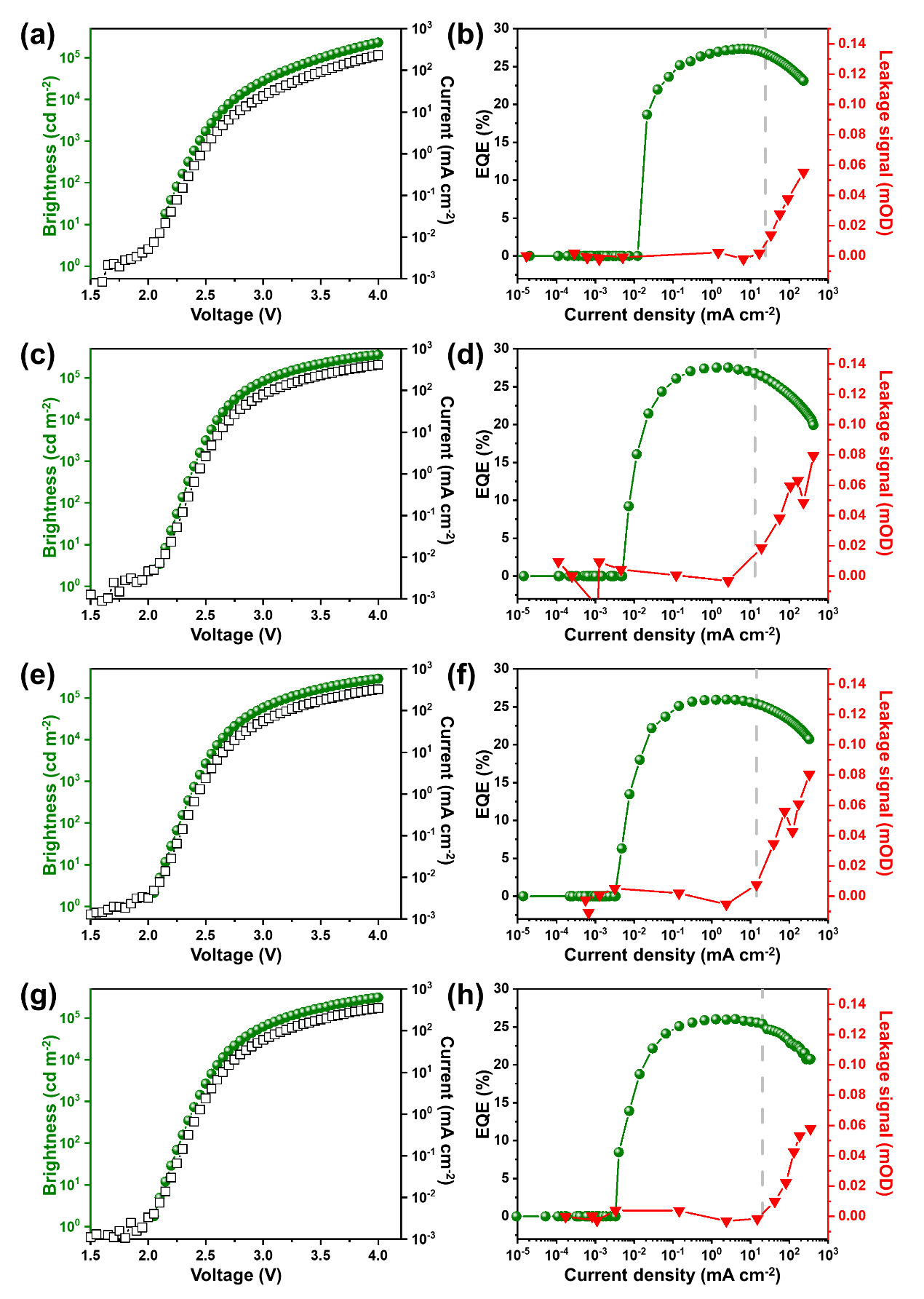


**Figure S18.** The left panels display the luminescence-current-voltage curves for same other QLEDs investigated in this study. The corresponding right panel plot the EQE alongside the amplitude of leakage signal in the same graph.


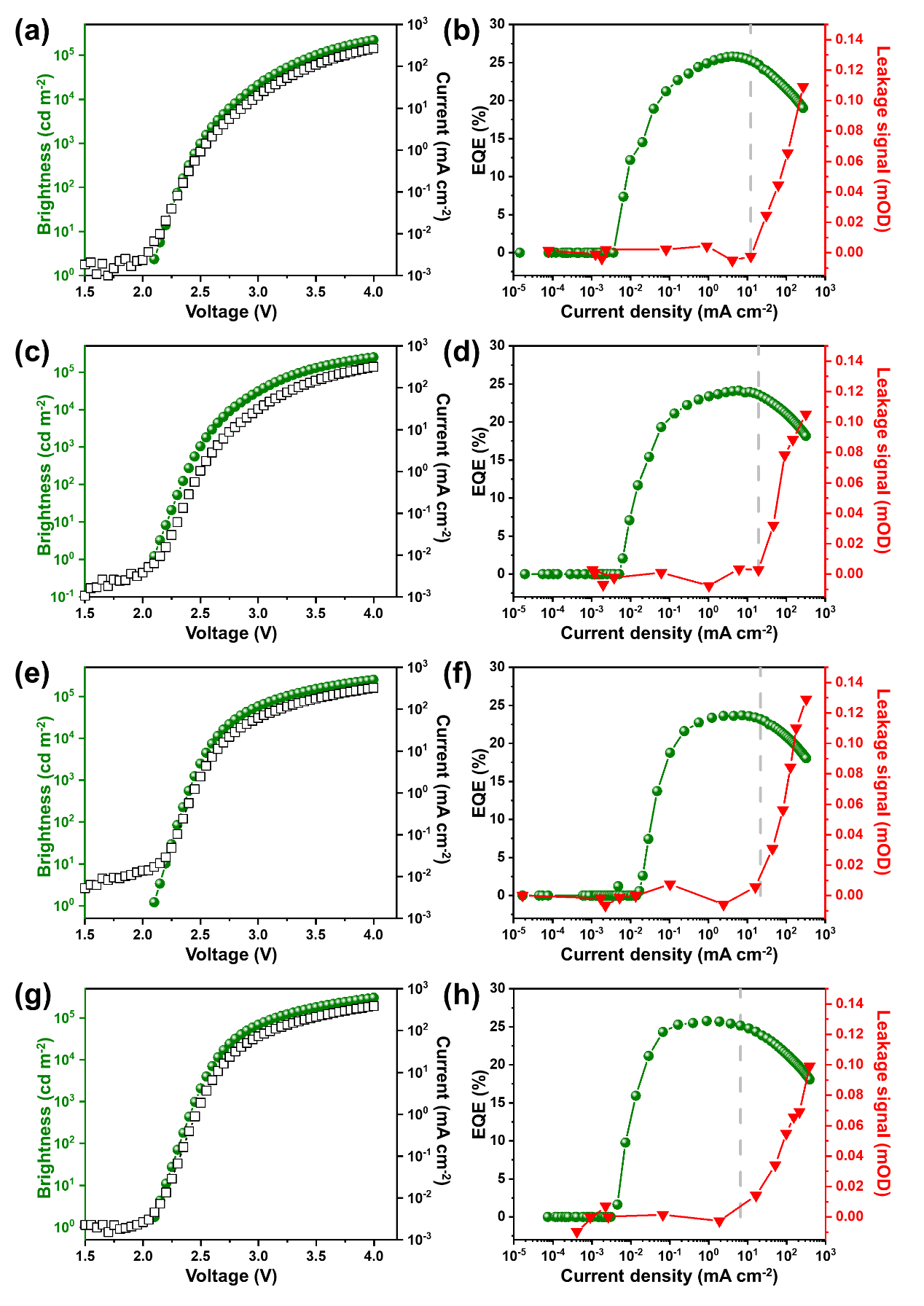


**Figure S19.** Continue of Figure S18.


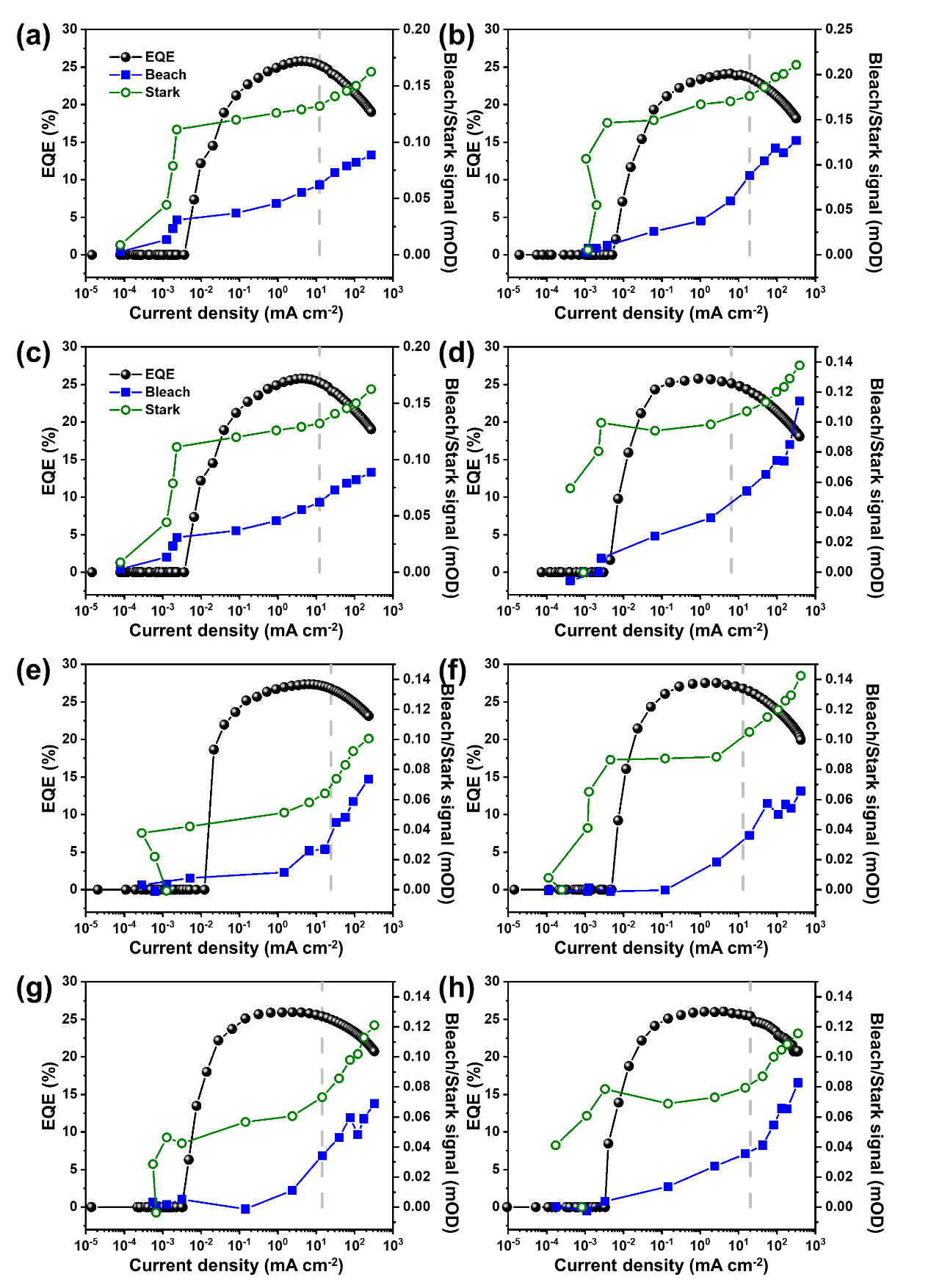


**Figure S20.** The amplitudes of bleach and Stark signal, along with EQE, as a function of current density for eight different green QLEDs.

**
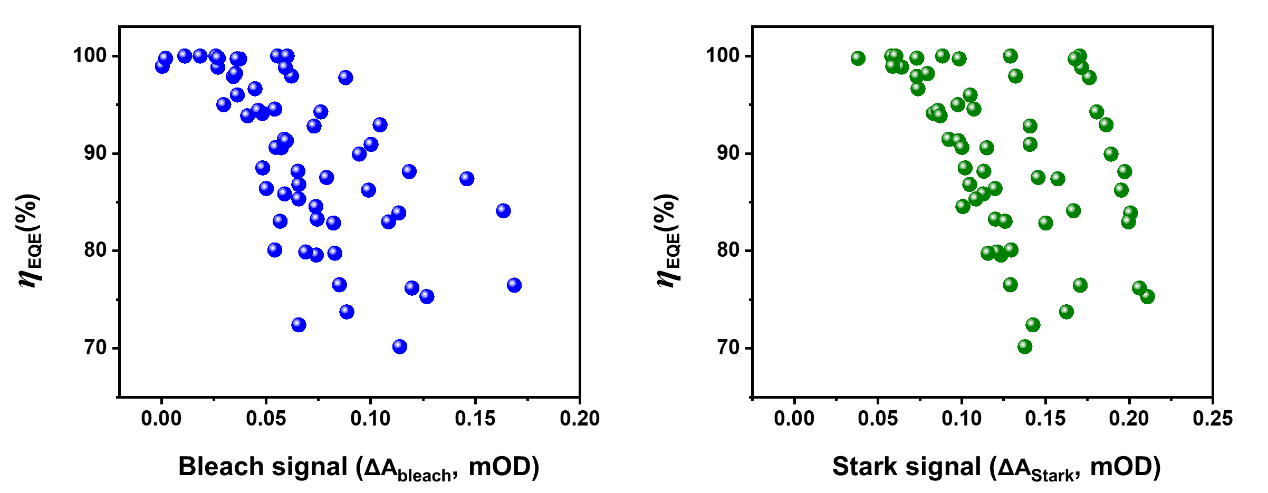
**

**Figure S21.** The correlation between the amplitudes of bleach and Stark signal and *η*_EQE_, based on statistical analysis of nine QLED devices. Each QLED contributes multiple data points at varying current densities. No clear correlation is observed.


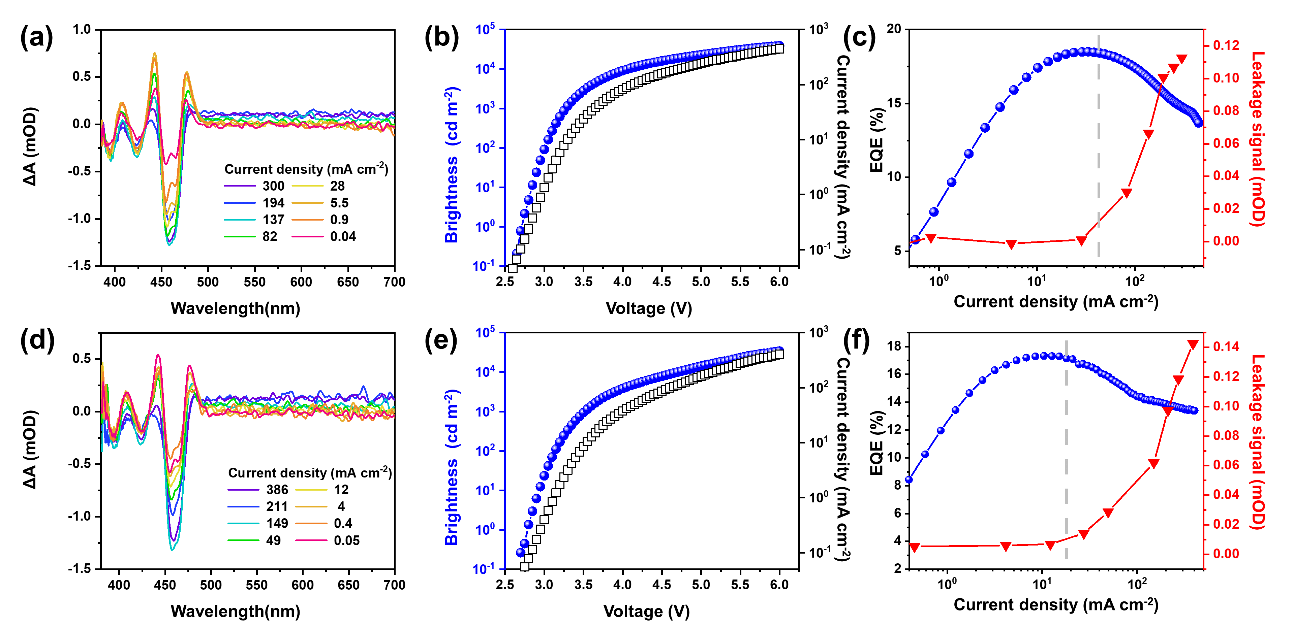


**Figure S22.** **Study of the correlation between electron leakage and roll-off in two blue QLEDs.** (a) and (d) Equilibrium E-TA spectra at different current densities, with T_d_ > 8 μs. (b) and (e) The brightness and current density of blue QLEDs as a function of voltage. (c) and (f) ΔA_leakage_ and EQE as a function of current density. Panels (a)–(c) show data from one blue QLED, while panels (d)–(f) correspond to data from the other.

**References**

[1] D. Ompong, J. Singh, *physica status solidi c* **2016**, 13, 89.

[2] D. Beljonne, Z. Shuai, G. Pourtois, J. L. Bredas, *The Journal of Physical Chemistry A* **2001**, 105, 3899.

[3] B. Kraabel, D. Moses, A. J. Heeger, *The Journal of Chemical Physics* **1995**, 103, 5102.

[4] S. Chen, W. Cao, T. Liu, S.-W. Tsang, Y. Yang, X. Yan, L. Qian, *Nature Communications* **2019**, 10, 765.

[5] A. Sacra, D. J. Norris, C. B. Murray, M. G. Bawendi, *The Journal of Chemical Physics* **1995**, 103, 5236.

[6] J. Huang, Z. Huang, Y. Yang, H. Zhu, T. Lian, *Journal of the American Chemical Society* **2010**, 132, 4858.

[7] X. Yan, C. Chen, B. Wu, F. Sun, H. Bao, W. Tian, S. Chang, H. Zhong, S. Jin, *The Journal of Physical Chemistry Letters* **2024**, 15, 8593.

[8] Y. Deng, X. Lin, W. Fang, D. Di, L. Wang, R. H. Friend, X. Peng, Y. Jin, *Nature Communications* **2020**, 11, 2309.

[9] A. Zabet-Khosousi, A.-A. Dhirani, *Chemical Reviews* **2008**, 108, 4072.
